# Modeling Physical Activity Profiles in COPD Patients: A Basis Expansion Approach to Variable Domain Functional Regression Models

**DOI:** 10.1002/bimj.70164

**Published:** 2026-08-27

**Authors:** Pavel Hernández‐Amaro, María Durbán, M. Carmen Aguilera‐Morillo, Cristobal Esteban Gonzalez, Inmaculada Arostegui

**Affiliations:** ^1^ Universidad Carlos III de Madrid Madrid Spain; ^2^ Universitat Politècnica de València Valencia Spain; ^3^ Osakidetza Basque Health Service Bilbao Spain; ^4^ University of the Basque Country UPV/EHU Bilbao Spain

**Keywords:** B‐splines, COPD, mixed models, variable domain functional data

## Abstract

Physical activity plays an important role in the well‐being of patients with Chronic Obstructive Pulmonary Disease (COPD). The telEPOC program, developed at the Galdakao‐Usansolo University Hospital (Vizcaya, Spain), provided a telemonitoring data set resulting in a variable domain functional data set. In this sense, the aim is to study the impact of physical activity (measured as daily steps collected in a time interval delimited by the patient's participation in the experiment) on the rate of hospitalizations. To address this challenge, a novel basis expansion approach is proposed to fit a variable‐domain functional regression model, which assumes the functional nature of the covariate (daily steps) and incorporates the sample data into the model without alignment. This will enrich the interpretability of the model by incorporating the information provided by the domain through a two‐dimensional regression coefficient. In addition, a penalized estimation of the model is proposed in terms of a 2D P‐spline penalty, which allows considering different degree of smoothness for each dimension of the functional regression coefficient (anisotropic penalty). The performance of our methodology is also tested and compared with state‐of‐the‐art methods by means of a simulation study. Software for our method can be found in the **VDPO** R package available in CRAN.

## 1 Introduction

Functional data are usually found as discrete and often noisy observations of the true underlying function, measured at different locations in time, space, or other continuums. The domain where the data are observed is usually assumed to be the same across observations. Functional data where the domain is not the same for all the observations are named variable domain functional data. This type of data can be found in many data sets and a variety of research fields like biology (Kulbaba et al. 2017), agriculture (Panayi et al. 2017), medicine (Gaynanova et al. 2022), among others.

**FIGURE 1:**
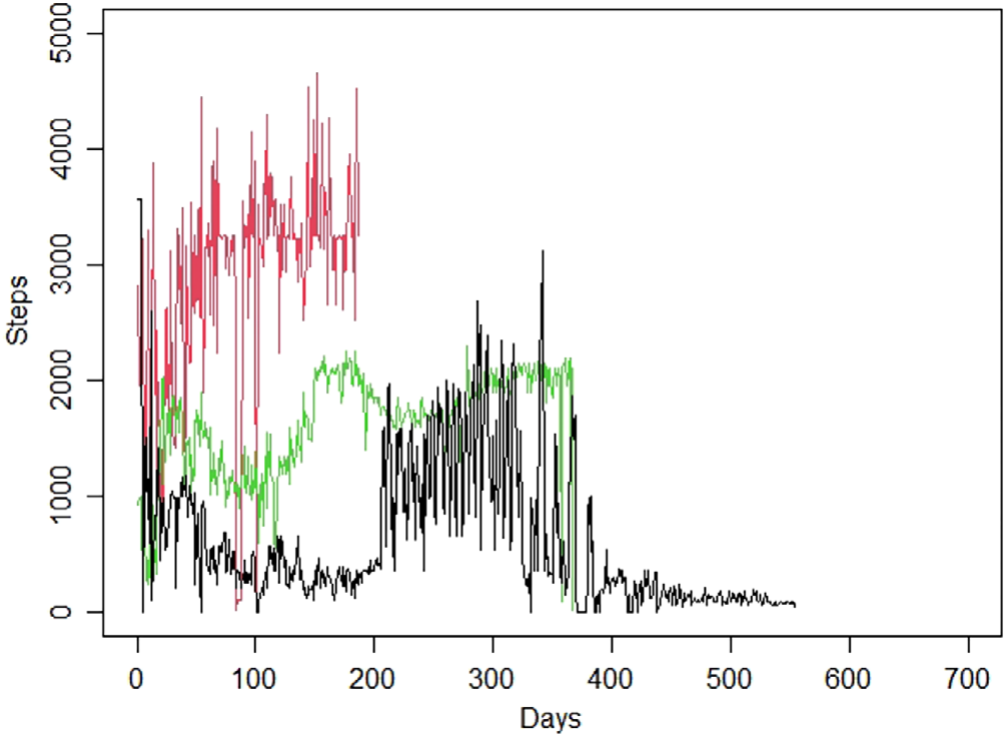
Daily steps of three different patients of the telEPOC program.

Our particular motivation is the telEPOC program (Esteban et al. 2016). In this study, a wide range of data from 119 patients suffering from Chronic Obstructive Pulmonary Disease (COPD) is collected, being the most important, the physical activity performed by each patient. The physical activity is measured as daily steps, with the particularity that the number of days where steps are collected differs from patient to patient, varying from 64 days up to 1287 days, as shown in Figure 1. There are two major reasons for this: (i) the exact day of sign‐up in the study is different from patient to patient (the inclusion dates go from May 31, 2010 to December 7, 2013) and (ii) the time each patient spends in the study is different, being one of the causes that 21 patients died during the study period. Therefore, we are in presence of a variable domain functional data set.

One of the goals of the telEPOC program is to determine how physical activity affects health in COPD patients. More precisely, the aim is to estimate the relationship between the number of hospitalizations due to COPD (scalar discrete r.v.) and physical activity (functional covariate).

There are some studies that showed a relationship between physical activity and hospitalization for exacerbation of COPD (Garcia‐Aymerich et al. 2006; Garcia‐Rio et al. 2012). Moreover, the change in the level of physical activity was linked to the change in hospitalizations in COPD patients (Esteban et al. 2014). These studies were cross‐sectional or longitudinal, observational, or epidemiological. Physical activity was measured by questionnaires or by accelerometers and the tendency of the results was the same in all of them. The measurements were carried out at baseline or from time to time (e.g., yearly), hence, several events could have happened in that period, that could influenced the change of the level of physical activity. In a telemonitoring program (telEPOC) the information has to be more frequent, almost continuous because decisions depend on it. In other words, having daily information about physical activity is a key element in the early stages of the “decision‐making” process.

Therefore, we are posed with a regression problem where the response variable Y (number of hospitalizations) is a scalar and the predictor X (daily steps) is a function whose values vary over a continuous domain of varying length among subjects, that is, $\left\{X_{i}\left(t\right):t\in \left[d_{i},T_{i}\right],\;i=1,\text{…},N.\right\}$. Essentially, this means that every curve can have observation points that fall in different domains. In our case study, the data can be left‐aligned and ordered without affecting the information of the results, having for all the data the same initial point $t\in [0,T_{i}]$ and $T_{i}\leq T_{i+1}\;\forall i$.

To deal with the variable domain functional data, Gellar et al. (2014) introduced the variable domain functional regression (VDFR) model, introducing the domain information in the formulation of the functional model. This approach works directly with the raw data without considering the functional nature of the covariates and imposes an isotropic penalty in the model estimation.

As an alternative, a basis expansion approach for a variable domain functional regression (BE‐VDFR) model is proposed. The main contributions of the proposed methodology are as follows: it restores the original functional nature of the data by recovering smooth latent trajectories from noisy, discretely observed sample curves, which is particularly advantageous in scenarios involving sparse or irregularly sampled functional covariates—settings for which no existing variable‐domain method is currently applicable. In addition, the model incorporates a highly flexible representation of the functional coefficient through the use of B‐spline bases combined with anisotropic penalization. This allows for differential control of smoothness across dimensions, enabling the model to adapt to complex patterns in the data while maintaining interpretability and computational tractability

In the next section, we explain the main problem of modeling variable domain functional data and show the state of the art in this regard. In Section 3, the BE‐VDFR model is explained, with the corresponding estimation procedure. In Section 4, a simulation study is carried out to evaluate the performance of the proposed method in comparison with the methods introduced in Section 2 highlighting the outperformance of the proposed methodology. In Section 5, the results of applying the proposed methodology to the telEPOC program are shown. Finally, a discussion is provided in Section 6.

## 2 Variable Domain Functional Regression

In most of functional data problems, the functional predictor is assumed to have a common domain for all sample units. In this context, the sample information is usually given by $\left\{Y_{i},X_{i}\left(t\right),C_{i}\right\}$, i=1,…,N, where $C_{i}$ is a vector of nonfunctional covariates, $Y_{i}$ is a scalar outcome following an exponential family distribution with mean $\mu_{i}$, and $\left\{X_{i}\left(t\right):t\in T\right\}$ is the functional predictor. From this information, the functional generalized linear model is given by

(1) $$\eta_{i}=g\left(\mu_{i}\right)=\alpha +C_{i}\gamma +\int_{0}^{T}X_{i}\left(t\right)\beta \left(t\right)\;dt,$$

where g(·) is the corresponding link function. This model, named scalar‐on‐function (SOF) regression model, was one of the first regression models extended to the case of functional data. The main theoretical aspects related to this model were studied in Cardot et al. (1999) and in James (2002), in the more general framework of generalized linear models. This model has been widely used in the literature, leading to numerous applications and new methodological developments. Penalized versions of the functional generalized linear models can be seen in works, such as Aguilera‐Morillo et al. (2013); Cardot and Sarda (2005); Goldsmith et al. (2011), among others.

This methodology is highly effective for modeling functional data when the domain remains consistent across observations. However, for functional data with variable domains, it is necessary to first transform the sample curves. A common approach is to register the sample curves to a common domain. However, this additional step has certain drawbacks with variable‐domain functional data: important information related to the specific shape of the curves may be lost, introducing bias in curve estimation and resulting in functional coefficients that are harder—or even impossible—to interpret. In addition, this registration process can be computationally expensive in some cases. For further insights on curve registration, see Ramsay and Silverman (2005) and Ramsay et al. (2009).

In data sets with variable domains, the length of the sample paths may hold valuable information. Thus, incorporating this aspect into the functional regression model is essential for effectively managing this type of data. To address the limitations of the SOF model for variable‐domain functional data, Gellar et al. (2014) proposed a new approach: the VDFR model, given by

(2) $$\eta_{i}=g\left(\mu_{i}\right)=\alpha +C_{i}\gamma +\frac{1}{T_{i}}\int_{0}^{T_{i}}X_{i}\left(t\right)\beta \left(t,T_{i}\right)\;dt,\;\;t\in \left[0,T_{i}\right].$$

In this model, the univariate coefficient function β(t) is replaced by a bivariate coefficient function β(t,T), which now depends on both the time instant t and the data domain T. This functional coefficient is represented as a surface, where curves obtained by fixing $T=T_{i}$ represent the optimal function for $X_{i}\left(t\right)$ to express its contribution to $g(\mu_{i})$. Furthermore, the integration limits, which were previously fixed from 0 to T, are now subject‐specific, eliminating the need for prior curve registration.

The authors work directly with the discrete observations $x_{ik}$ of each sample curve $X_{i}\left(t\right)$, collected at a finite set of observation points $\left\{t_{ik}:k=0,\text{…},m_{i}\right\}$, disregarding the functional nature of the curves. As a result, the methodology proposed by Gellar et al. (2014) can be classified as a “partially functional approach” because it does not account for the functional form of the variable X(t). Instead, it operates directly on the raw data matrix, consisting of sample curves at discrete observation points. This approach sacrifices the opportunity to recover the true functional form of the data, missing the chance to filter out noise commonly present in discrete observations, which subsequently introduces errors into the model coefficients.

In addition, this approach recommends using thin‐plate spline functions as the basis for representing the functional coefficient, with an isotropic penalization that enforces the same degree of smoothness across both dimensions of the coefficient surface. This may lead to biased estimates of the functional coefficient, as demonstrated in the simulation section.

## 3 Basis Expansion Approach

As we mentioned in the previous section, partially functional approaches work on the discrete observations $x_{ij}$ of each sample curve $X_{i}\left(t\right)$ at a set of points $\left\{t_{ik},\;k=0,\text{…}T_{i}\right\}$. However, in practice, it is very common to find functional data sets observed with error or noise. In this section, a basis expansion approach is proposed by performing a presmoothing of the sample curves, recovering the smooth functional form of the data by means of a basis representation of the sample curves. A review on the different ways to estimate the basis coefficients as well as the different penalties used and their performance can be seen in Aguilera and Aguilera‐Morillo (2013). In addition to the basis representation of the sample curves, a basis expansion of the functional coefficient is also assumed, providing, as a consequence, a new formulation of the VDFR model.

### 3.1 Model Formulation in Terms of Basis Functions

Let Y be the scalar response variable and X(t) the functional predictor. Let X(t) be a second‐order continuous‐time stochastic process, with sample functions $\left\{X_{i}\left(t\right):t\in \left[d_{i},T_{i}\right],\;i=1,\text{…},N\right\}$ in the Hilbert space $H_{1}=L^{2}\left(T\right)$ of square integrable functions, with the usual inner product:

$$<f,g>=\left\{f:T\leftarrow R:\int_{T}f^{2}\left(t\right)\;dt<\infty \right\}.$$

Assuming a basis representation for both the sample curves and the functional coefficient of model (2), we have

$$\begin{array}{ccc} X_{i}\left(t\right) & = & \sum_{j=1}^{p_{i}}a_{ij}\phi_{j}\left(t\right)=\phi_{i}^{\prime }\left(t\right)a_{i},\\ \beta (t,T) & = & \sum_{l=1}^{q}\sum_{k=1}^{r}b_{lk}\varphi_{l}\left(t\right)\psi_{k}\left(T\right)=M\left(t,T\right)b, \end{array}$$

where $\phi_{i}\left(t\right)={\left(\phi_{1}\left(t\right),\phi_{2}\left(t\right),\text{…},\phi_{p_{i}}\left(t\right)\right)}^{\prime }$ and M(t,T) are the basis used in the representation of the functional data and the functional coefficient, respectively, and $a_{i}$ and b their respective basis coefficients. Note that M(t,T) is a bivariate basis function formed from the tensor product of φ(t) and ψ(T), with $\psi \left(T\right)={\left(\psi_{1}\left(T\right),\psi_{2}\left(T\right),\text{…},\psi_{r}\left(T\right)\right)}^{\prime }$ and $\varphi \left(t\right)={\left(\varphi_{1}\left(t\right),\varphi_{2}\left(t\right),\text{…},\varphi_{q}\left(t\right)\right)}^{\prime }$.

Here, $p_{i}$, q, and r are the numbers of basis functions for $\phi_{i}\left(t\right)$, φ(t), and ψ(T), respectively. For simplicity, we assume a common number of basis functions p for representing all sample curves, that is, $p_{i}=p$ and $\phi_{i}\left(t\right)=\phi \left(t\right)$ for all i=1,…,N, though this can be relaxed.

The choice of basis functions is important and often data‐driven: if the data exhibit periodic trends, a Fourier basis may be appropriate; if the data display strong local behavior and derivatives are unimportant, wavelet bases are common. In this paper, we use B‐spline bases (De Boor 2001), which are often chosen when the underlying signal is assumed to be smooth and derivatives up to a certain order are needed.

Assuming a basis representation for both the sample curves and the functional coefficient, model (2) becomes the following multivariate regression model:

(3) $$\begin{array}{c} \eta =\alpha +C\gamma +\frac{1}{T}\int_{0}^{T}X\left(t\right)\beta \left(t,T\right)\;dt=\alpha +C\gamma +A\Psi b=B\theta , \end{array}$$

where T represents the vector of curve lengths, allowing each sample curve to have different integration limits.

The matrix of coefficients A is block diagonal, where the ith block of the diagonal is the estimated vector of basis coefficients $a_{i}^{\prime }$. To estimate these basis coefficients, we follow Aguilera and Aguilera‐Morillo (2013); using penalized least squares regression with a discrete penalty based on second‐order differences between adjacent B‐spline coefficients (Eilers and Marx 1996). Finally, the matrix of inner products Ψ is a block column matrix of weighted inner products, with the ith block being a weighted inner product between the basis ϕ(t) and $M(t,T_{i})$, that is, $\Psi_{Np\times qr}={\left(\Psi_{1},\text{…},\Psi_{N}\right)}^{\prime }$, where $\Psi_{i}=\frac{1}{T_{i}}\left\langle \phi \left(t\right),M\left(t,T_{i}\right)\right\rangle$.

(4) $$\begin{array}{cc} A={\left(\begin{array}{cccc} a_{1}^{\prime } & 0 & \text{…} & 0\\ 0 & a_{2}^{\prime } & 0 & \text{…}\\ \text{…} & \text{…} & \text{…} & \text{…}\\ 0 & 0 & \text{…} & a_{N}^{\prime } \end{array}\right)}_{N\times Np} & \Psi ={\left(\begin{array}{c} \frac{1}{T_{1}}\int_{0}^{T_{1}}\phi_{1}\left(t\right)M\left(t,T_{1}\right)\;dt\\ \frac{1}{T_{2}}\int_{0}^{T_{2}}\phi_{2}\left(t\right)M\left(t,T_{2}\right)\;dt\\ \vdots \\ \frac{1}{T_{N}}\int_{0}^{T_{N}}\phi_{N}\left(t\right)M\left(t,T_{N}\right)\;dt \end{array}\right)}_{Np\times qr}. \end{array}$$

The basis representation of curves adds complexity, as it requires calculating the inner product between a univariate and a bivariate function. This issue was recently addressed by Masak et al. (2023), via the definition of partial inner product defined as:

The integrals in (4) are numerically approximated by the Composite Simpson method. The challenge is to perform integration only over t while preserving the bivariate structure of the basis $M(t,T_{i})$. To address this, in each integration step, we construct the matrix $M_{i}$ as a Kronecker product of matrices $\varphi_{i}$ and $\psi_{i}$. The matrix $\psi_{i}$ is the result of evaluating the basis ψ(T) in the corresponding domain $T_{i}$, that is, $\psi_{i}=\psi \left(T_{i}\right)$, and matrix $\varphi_{i}$ is the result of evaluate the basis φ(t) in a set of points determined by the integration method (this set of points change according with the domain of every curve). The basis ϕ(t) is evaluated in the same set of points as the basis φ(t) in every iteration resulting in a matrix $\phi_{i}$. Finally, when the matrix $M_{i}$ is recalculated, the product between $M_{i}$ and $\phi_{i}$ is performed. The previous procedure is summarized in the following algorithm 1:

ALGORITHM 1 **Calculation of the partial inner product**

**Table:** 

**Input:** $T_{i}$ ‐ Domain length for curve i	
φ(t) ‐ First basis function for functional coefficient β(t,T) representation	
ψ(T) ‐ Second basis function for functional coefficient β(t,T) representation	
ϕ(t) ‐ Basis function for functional data representation	
n ‐ Number of points for numerical integration	
**Output:** $\Psi_{i}$: Partial inner product of $\phi_{i}\left(t\right)$ and $M(t,T_{i})$	
**Steps:**	
1:	Generate integration points using Composite Simpson's method:
	$t_{\text{points}}\leftarrow \text{CompositeSimpson}\left(0,T_{i},n\right)$
2:	Evaluate basis function ψ(T) at domain $T_{i}$:
	$\psi_{i}\leftarrow \psi \left(T_{i}\right)$
3:	Evaluate basis functions $\phi_{i}\left(t\right)$ and φ(t) at integration points $t_{\text{points}}$: $\varphi_{t}\leftarrow \varphi \left(t_{\text{points}}\right)$ $\phi_{it}\leftarrow \phi \left(t_{\text{points}}\right)$
4:	Calculate $M_{i}$ using Kronecker product:
	$M_{i}\leftarrow \varphi_{t}⊗\psi_{i}$
5:	Compute final product:
	$\Psi_{i}\leftarrow \phi_{it}^{\prime }M_{i}$

The function CompositeSimpson($0,T_{i},n$) calculates the evaluation points needed to integrate from 0 to $T_{i}$ using the Composite Simpson method, as outlined in Burden et al. (2015). Note that the matrix $\psi_{i}$ represents the ith row of a broader matrix $\psi \in R^{N\times r}$, which is linked to all the distinct domains present in the data set, that is, $T=[T_{1},\text{…},T_{N}]$. For each subject i, the corresponding row is selected from this matrix.

It is also possible for two or more sample curves to share the same domain; in such cases, the same row of the matrix ψ will be assigned to each of these curves.

### 3.2 Model Estimation Through a Mixed‐Model Representation

The multivariate regression model (3) falls within the category of generalized linear models, allowing parameter estimation through the maximum likelihood method. In our motivational example, the response variable follows a Poisson distribution with likelihood:

$$L\left(\theta ,y\right)=\sum_{i=1}^{N}y_{i}\eta_{i}-\text{exp}\left\{\sum_{i=1}^{N}\eta_{i}\right\}.$$

Since the functional coefficient is represented using a B‐spline basis, the smoothness of the estimated coefficient depends on the basis dimension. To avoid the challenge of selecting the optimal number of basis functions, we adopt the penalized likelihood approach proposed by Eilers and Marx (1996), yielding the final penalized likelihood equation:

$$L_{p}\left(\theta ,y\right)=L\left(\theta ,y\right)-\frac{1}{2}\theta^{\prime }P\theta ,$$

where L(θ,y) is the likelihood of Y and P is the penalty term. Penalties are, in general, based on curve derivatives (Wood 2017) or differences between adjacent B‐splines coefficients (Eilers and Marx 1996). We employ the latter approach here.

Given that the functional parameter is two‐dimensional, an anisotropic two‐dimensional penalty is applied, allowing independent control of the functional coefficient's smoothness in each dimension. Then, the following penalty is added to the likelihood:

(5) $$P=\lambda_{t}\left(I_{r}⊗D_{t}^{\prime }D_{t}^{\prime }\right)+\lambda_{T}\left(I_{q}⊗D_{T}^{\prime }D_{T}\right),$$

where $I_{r}$ and $I_{q}$ are identity matrices of order r and q, respectively. The symbol ⊗ denotes the Kronecker product (Lee and Durbán 2011) and matrices $D_{t}$ and $D_{T}$ are second‐order differences matrices:

$$\begin{array}{ccc} D_{t} & = & {\left[\begin{array}{cccccc} 1 & -2 & 1 & 0 & \cdots & 0\\ 0 & 1 & -2 & 1 & \cdots & 0\\ 0 & 0 & 1 & -2 & \cdots & 0\\ \vdots & \vdots & \vdots & \vdots & \ddots & \vdots \\ 0 & 0 & 0 & 0 & \cdots & 1 \end{array}\right]}_{q-2\times q}\\ D_{T} & = & {\left[\begin{array}{cccccc} 1 & -2 & 1 & 0 & \cdots & 0\\ 0 & 1 & -2 & 1 & \cdots & 0\\ 0 & 0 & 1 & -2 & \cdots & 0\\ \vdots & \vdots & \vdots & \vdots & \ddots & \vdots \\ 0 & 0 & 0 & 0 & \cdots & 1 \end{array}\right]}_{r-2\times r}. \end{array}$$

This penalized approach avoids the need to carefully choose the number of basis functions and position of the knots, provided that the basis size is sufficiently large. Instead, smoothness is controlled by the parameters $\lambda_{t}$ and $\lambda_{T}$.

We estimate the parameters of the BE‐VDFR model using a mixed‐model reparameterization of the penalized spline. This transformation enables simultaneous estimation of all model parameters, including the smoothing parameters. A brief description of this reparameterization follows to facilitate understanding of the methodology. For a comprehensive explanation of the mixed‐model reparameterization of a penalized spline, see Lee (2010). Our goal is to transform

(6) η=Bθ⇒Xν+Zδ,δ∼N(0,G),

where X and Z are the model matrices, ν and δ are fixed and random effects, respectively, and G is the variance–covariance matrix of the random effects, which depends on the variance components $\tau_{t}^{2}$ and $\tau_{T}^{2}$ that are related to the smoothness parameters as $\tau_{t}^{2}=\frac{1}{\lambda_{t}}$ and $\tau_{T}^{2}=\frac{1}{\lambda_{T}}$ (Brumback et al. 1999). This reparametsrization uses a transformation matrix L based on the Singular Value Decomposition (SVD) of the product of the difference matrices $D_{i}^{\prime }D_{i}$. Let

$$D_{i}^{\prime }D_{i}=\left[U_{in}|U_{is}\right]\left[\begin{array}{cc} 0_{2} & \\ & {\tilde{\Sigma }}_{i} \end{array}\right]\left[\begin{array}{c} U_{in}^{\prime }\\ U_{is}^{\prime } \end{array}\right]$$

be the SVD of $D_{i}^{\prime }D_{i}$, for i={t,T}, where $U_{in}$ and $U_{is}$ are eigenvectors corresponding to zero and nonzero eigenvalues, respectively, while $0_{2}$ and ${\tilde{\Sigma }}_{i}$ are squared matrices with the zero and nonzero eigenvalues on its diagonal, respectively. The transformation matrix L is defined as $L=\left[L_{n}|L_{s}\right]=\left[U_{Tn}⊗U_{tn}|U_{Ts}⊗U_{tn}:U_{Tn}⊗U_{ts}:U_{Ts}⊗U_{ts}\right]$, where $L_{n}$ and $L_{s}$ are submatrices of the transformation matrix L related to the eigenvectors associated with the zero and nonzero eigenvalues, respectively. These matrices will be linked with the fixed and random effects, respectively, ensuring that only the random effects are penalized.

Alternative transformation matrices are possible, but our choice enables recovery of the estimated parameter $\hat{\theta }$ from the mixed‐model coefficients, due to the orthogonality of L, allowing for the functional coefficient $\hat{\beta }\left(t,T\right)$ to be recovered. Using this matrix, model (3) is reparameterized as follows: $\eta =B\theta =BLL^{\prime }\theta =X\nu +Z\delta$, where $BL=\left[BL_{n}|BL_{s}\right]=\left[X|Z\right]$, and the variance–covariance matrix G is obtained by transforming the original penalty as $G^{-1}=L^{\prime }PL$:

(7) $$\begin{array}{c} G^{-1}=\left(\begin{array}{ccc} \frac{1}{\tau_{T}^{2}}{\tilde{\Sigma }}_{T}⊗I_{2} & & \\ & \frac{1}{\tau_{t}^{2}}I_{2}⊗{\tilde{\Sigma }}_{t} & \\ & & \frac{1}{\tau_{T}^{2}}{\tilde{\Sigma }}_{T}⊗I_{q-2}+\frac{1}{\tau_{t}^{2}}I_{r-2}⊗{\tilde{\Sigma }}_{t} \end{array}\right), \end{array}$$

where $I_{2}$, $I_{q-2}$, $I_{r-2}$ are identity matrices of order 2, q−2, and r−2, respectively. Once the model is transformed, penalized quasi‐likelihood (Breslow and Clayton 1993) is used to estimate the mixed‐model coefficients and variance components. In practice, the SOP algorithm (Rodríguez‐Álvarez et al. 2019) to speed up computations.

### 3.3 Uncertainty Quantification

To quantify the uncertainty of the estimate of $\hat{\beta }\left(t,T\right)=M\left(t,T\right)\hat{b}$, we need:

(8) $$\begin{array}{c} \mathrm{Var}\left(\hat{\beta }\left(t,T\right)\right)=\mathrm{Var}\left(M\left(t,T\right)\cdot \hat{b}\right)=M\left(t,T\right)\cdot \mathrm{Var}\left(\hat{b}\right)\cdot M^{\prime }\left(t,T\right), \end{array}$$

where $\hat{b}$ represents a submatrix of the variance–covariance matrix of the model coefficients $\hat{\theta }$, since $\theta^{\prime }=\left(\alpha ,\gamma ,b\right)$. Although we do not directly estimate $\hat{\theta }$, we estimate its transformed counterpart through the matrix L. Due to the orthogonality of L, it is possible to recover $\hat{\theta }$ from the transformed coefficients ${\hat{\omega }}^{\prime }={\left[\hat{\nu }:\hat{\delta }\right]}^{\prime }$, as defined in Equation (6):

(9) $$L^{\prime }\hat{\theta }=\hat{\omega }\Rightarrow L\hat{\omega }=\hat{\theta }\Rightarrow \mathrm{Var}\left(L\hat{\omega }\right)=L\cdot \mathrm{Var}\left(\hat{\omega }\right)\cdot L^{\prime }=\mathrm{Var}\left(\hat{\theta }\right).$$

This result allows us to express the variance–covariance matrix of $\hat{\theta }$ in terms of the variance–covariance matrix of the transformed coefficients, facilitating the computation of confidence intervals for $\hat{\theta }$. The construction of confidence intervals for the curves corresponding to a particular domain $\hat{\beta }$ are easily obtained from the corresponding row of $\mathrm{Var}(\hat{\beta }\left(t,T\right))$, or can be directly calculated as $\mathrm{Var}(\hat{\beta }\left(t,T_{i}\right))$.

The developed methodology has been implemented in the form of an R package for RStudio (R Core Team 2013) which has been accepted in CRAN with the name of **VDPO**.

## 4 Simulation Study

To evaluate the performance of the BE‐VDFR model, we conducted a comprehensive simulation study in this section, comparing the outcomes with those from both the VDFR model and the standard SOF regression models. For the SOF model, a preliminary registration of the curves was performed to standardize input. In the case of the VDFR model, we adopt the version based on the thin plate regression spline (TPRS) bases with isotropic penalties and untransformed functions. This choice is motivated to ensure a fair evaluation under settings where domain standardization offers limited benefit and the original time scale preserves interpretability, particularly given that all domains in our simulations are sufficiently long and consistently sampled.

### 4.1 Simulation Scenarios

For simplicity, only models with one functional covariate and no nonfunctional covariates have been considered. We simulated 100 data sets for each combination of the parameters described below, resulting in a total of 32 distinct scenarios:

- **Outcome types**: Two types of outcomes were considered, continuous and count data, both modeled with the following linear predictor:
$$\eta_{i}=\frac{1}{T_{i}}\sum_{t=1}^{T_{i}}X_{i}\left(t\right)\beta \left(t,T_{i}\right),\;\;t=1,\text{…},T_{i}\leq 100,$$
  where $Y_{i}=\eta_{i}+\varepsilon_{i}$ with $\varepsilon_{i}∼N\left(0,1\right)$ for continuous outcomes, and $Y_{i}∼\text{Poisson}\left(\mu_{i}\right)$ with $\mu_{i}=\exp\left(\eta_{i}\right)$ for count data.
- **Data domain distributions**: Two distributions were considered for $T_{i}$: Uniform ($T_{i}∼U\left(10,100\right)$) and Negative Binomial ($T_{i}∼\text{NegBin}\left(1,p=0.04\right)$). For both cases, the sample curves were limited to between 10 and 100 observations (we considered 10 to be an acceptable minimum to consider the observed points as observation of the true underlying functional data $X_{i}\left(t\right)$). In the Negative Binomial case, values outside this range were truncated to ensure a minimum of 10 and a maximum of 100 observations.
- Two different distribution for the data domain $T_{i}$: Uniform ($T_{i}∼U\left(10,100\right)$) and Negative Binomial ($T_{i}∼NegBin\left(1,p=0,04\right)$).
- **Functional covariate simulation**: The true functional covariate $X_{i}\left(t\right)$ was generated as follows:
$$\begin{array}{ccc} X_{i}\left(t\right) & = & u_{i}+\sum_{k=1}^{10}\left\{v_{ik1}\cdot sen\left(\frac{2\pi k}{100}t\right)+v_{ik2}\cdot \cos\left(\frac{2\pi k}{100}t\right)\right\}\\ & & +\delta_{i}\left(t\right), \end{array}$$
  where $u_{i}∼N\left(0,1\right)$, $v_{ik1},v_{ik2}∼N\left(0,\frac{4}{k^{2}}\right)$, and $\delta_{i}\left(t\right)∼N\left(0,\sigma_{i}\right)$, with $\sigma_{i}=0.25\cdot \text{Var}\left(X_{i}\left(t\right)\right)$. This ensures that the variability of the noise added to each covariate is one‐fourth of its variance it is consistent across curves.
- **Sample sizes**: Two sample sizes were evaluated, N={100,200}.
- **Functional coefficient configurations**: Four configurations for the functional coefficient β(t,T) were defined as follows:

 

$$\begin{array}{ccc} & & \beta_{1}\left(t,T_{i}\right)=10\frac{t}{T_{i}}-5\\ & & \beta_{2}\left(t,T_{i}\right)=\left(1-\frac{2T_{i}}{T_{N}}\right)\times \left(5-40{\left(\frac{t}{T_{i}}-0.5\right)}^{2}\right)\\ & & \beta_{3}\left(t,T_{i}\right)=5-10\left(\frac{T_{i}-t}{T_{N}}\right)\\ & & \beta_{4}\left(t,T_{i}\right)=sen\left(\frac{2\pi T_{i}}{T_{N}}\right)\times \left(5-10\left(\frac{T_{i}-t}{T_{N}}\right)\right), \end{array}$$

where $T_{N}=\max\left\{T_{1},\text{…},T_{N}\right\}$.

For the BE‐VDFR model, 30 cubic B‐splines (p=30) were used to represent the functional data while for the representation of the functional coefficient the number of basis functions for both the marginal basis has been 50 and 30, respectively (q=50,r=30), creating a two‐dimensional basis of size 1500. The SOF model utilized 25 cubic B‐splines for the representation of the functional data and the functional coefficient, respectively. For the BE‐VDFR and SOF model estimations, a second‐order differences matrix was applied as a penalty term. The VDFR model employed a thin plate basis of size 89 for the functional coefficient (the maximum size allowed by the software when $T_{N}=100$), with an isotropic penalty based on second‐order derivatives (as suggested by Gellar et al. 2014).

All simulations were carried out in RStudio (R Core Team 2013). The **SOP** package (Rodriguez‐Alvarez et al. 2021) was used for the reparameterization of the multivariate regression model and parameter estimation. The **refund** package (Goldsmith et al. 2021) was utilized for the SOF and VDFR model estimations, while the **VDPO** package was used for the BE‐VDFR model. The *R* script containing the simulation scenarios can be found at https://doi.org/10.5281/zenodo.21990643 while the simulation results are at https://doi.org/10.5281/zenodo.15741032.

### 4.2 Performance Criteria

We assess model performance across two key dimensions. The first is predictive accuracy, for which we employ a 10‐fold cross‐validation approach. The metric used to evaluate prediction errors is the mean of the Root Mean Square Error (RMSE) calculated for each fold:

$$RMSE_{j}=\sqrt{\frac{\sum_{i=1}^{N_{j}}{\left(Y_{ij}-{\hat{Y}}_{ij}\right)}^{2}}{N_{j}}},\;\;j=1,\text{…},10,$$

where $Y_{ij}$ represents the ith response variable in the jth fold, ${\hat{Y}}_{ij}$ is its corresponding estimate, and $N_{j}$ is the number of responses in the jth fold.

The second aspect evaluated is the model's ability to accurately estimate the functional coefficient β(t,T). For this, we use the Average Mean Square Error (AMSE) defined as follows:

$$AMSE^{r}=\frac{1}{T_{N}\left(T_{N}+1\right)}\sum_{k=10}^{T_{N}}\sum_{t=1}^{k}{\left\{\beta \left(t,k\right)-\hat{\beta }\left(t,k\right)\right\}}^{2},$$

where $T_{N}=\max\left\{T_{1},\text{…},T_{N}\right\}$ and $\hat{\beta }\left(t,k\right)$ represents the estimated functional coefficient.

It should be noted that with the SOF model, AMSE cannot be computed because this model assumes a single fixed domain for the functional coefficient.

The confidence bands of all the functional coefficients estimated with the proposed methodology have also been calculated.

### 4.3 Results

This section presents the results from the simulation study across all scenarios. The findings are summarized in tables, where Tables 1 and 2 provide the mean (standard deviation) AMSE and RMSE values for scenarios with a Normal‐distributed response variable, while Tables 3 and 4 present results for scenarios with a Poisson‐distributed response variable. Although a selection of plots is presented within this section, the complete set of graphical results is provided in the supplementary material.

**TABLE 1:** Mean and standard deviation (in parentheses) of 100 measures of AMSE when the response follows a Normal distribution.

Sample size	N=100				N=200			
Domain distribution	Uniform		Negative Binomial		Uniform		Negative Binomial	
Method	BE‐VDFR	VDFR	BE‐VDFR	VDFR	BE‐VDFR	VDFR	BE‐VDFR	VDFR
$\beta_{1}$	**0.00267**	0.0033	**0.0031**	0.00932	**0.000861**	0.00376	**0.000441**	0.00533
	$\left(4822\times 10^{-6}\right)$	$\left(5031\times 10^{-6}\right)$	$\left(4157\times 10^{-6}\right)$	$\left(2689\times 10^{-5}\right)$	$\left(2491\times 10^{-7}\right)$	$\left(9838\times 10^{-6}\right)$	$\left(3240\times 10^{-8}\right)$	$\left(2102\times 10^{-5}\right)$
$\beta_{2}$	**0.00359**	0.00361	**0.00639**	0.00924	**0.00296**	0.00583	**0.00562**	0.0112
	$\left(4826\times 10^{-6}\right)$	$\left(2566\times 10^{-6}\right)$	$\left(2056\times 10^{-6}\right)$	$\left(1198\times 10^{-5}\right)$	$\left(2091\times 10^{-7}\right)$	$\left(2736\times 10^{-6}\right)$	$\left(5036\times 10^{-8}\right)$	$\left(2359\times 10^{-5}\right)$
$\beta_{3}$	**0.00122**	0.00142	**0.00209**	0.00906	**0.000236**	0.00167	**0.0000581**	0.00169
	$\left(2067\times 10^{-6}\right)$	$\left(1422\times 10^{-6}\right)$	$\left(1355\times 10^{-5}\right)$	$\left(2786\times 10^{-5}\right)$	$\left(4364\times 10^{-8}\right)$	$\left(2320\times 10^{-6}\right)$	$\left(2357\times 10^{-9}\right)$	$\left(1523\times 10^{-6}\right)$
$\beta_{4}$	**0.000792**	0.00214	**0.00501**	0.00939	**0.00058**	0.00156	**0.000117**	0.00309
	$\left(3788\times 10^{-7}\right)$	$\left(1103\times 10^{-6}\right)$	$\left(3814\times 10^{-5}\right)$	$\left(2603\times 10^{-5}\right)$	$\left(8943\times 10^{-8}\right)$	$\left(9627\times 10^{-7}\right)$	$\left(1114\times 10^{-8}\right)$	$\left(8955\times 10^{-6}\right)$

**TABLE 2:** Mean and standard deviation (in parentheses) of 100 measures of RMSE when the response follows a Normal distribution.

Sample size	N=100						N=200					
Domain distribution	Uniform			Negative Binomial			Uniform			Negative Binomial		
Method	BE‐VDFR	VDFR	SOF	BE‐VDFR	VDFR	SOF	BE‐VDFR	VDFR	SOF	BE‐VDFR	VDFR	SOF
$\beta_{1}$	**0.174**	0.205	0.912	**1.36**	1.58	3.04	**1.63**	1.69	3.01	**1,32**	1,34	3,75
	(0.0012)	(0.0020)	(0.0115)	(0.0554)	(0.0935)	(0.3521)	(0.1017)	(0.1191)	(2.4822)	(0,0255)	(0,0187)	(0,5298)
$\beta_{2}$	**0.103**	0.116	0.685	**1.2**	1.2	3.34	**1.14**	1.15	2.04	**1,19**	1,2	4,08
	$\left(5971\times 10^{-4}\right)$	$\left(5791\times 10^{-4}\right)$	(0.0176)	(0.0070)	(0.0067)	(1.2165)	(0.0045)	(0.0056)	(0.1605)	(0,0042)	(0,0030)	(0,4622)
$\beta_{3}$	**0.17**	0.178	0.64	**6.77**	11.4	13.8	3.13	3.81	**2.05**	**9,43**	13,5	20,1
	(0.0010)	(0.0012)	(0.0079)	10.6587	(17.4251)	(22.8757)	(2.0918)	(5.4169)	(4.1549)	(17,1273)	(18,1327)	(30,7545)
$\beta_{4}$	**0.125**	0.16	0.668	**2.41**	3.19	3.45	**2.17**	2.46	5.44	**2.55**	3.35	3.31
	$\left(5508\times 10^{-4}\right)$	(0.0016)	(0.0196)	(0.5140)	(0.9112)	(2.1940)	(0.5157)	(0.9382)	(33.9579)	(0.3455)	(0.3761)	(0.8862)

**TABLE 3:** Mean and standard deviation (in parentheses) of 100 measures of AMSE when the response follows a Poisson distribution.

Sample size	N=100				N=200			
Domain distribution	Uniform		Negative Binomial		Uniform		Negative Binomial	
Method	BE‐VDFR	VDFR	BE‐VDFR	VDFR	BE‐VDFR	VDFR	BE‐VDFR	VDFR
$\beta_{1}$	**0.0211**	0.022	**0.0139**	0.0142	**0.0139**	0.0142	**0.00739**	0.0249
	$\left(2721\times 10^{-5}\right)$	$\left(4260\times 10^{-5}\right)$	$\left(1879\times 10^{-5}\right)$	$\left(4188\times 10^{-5}\right)$	$\left(1879\times 10^{-5}\right)$	$\left(4188\times 10^{-5}\right)$	$\left(5639\times 10^{-7}\right)$	$\left(2969\times 10^{-5}\right)$
$\beta_{2}$	**0.0155**	0.0166	**0.00985**	0.0108	**0.00414**	0.0263	**0.00689**	0.0189
	$\left(3926\times 10^{-5}\right)$	$\left(2094\times 10^{-5}\right)$	$\left(1209\times 10^{-5}\right)$	$\left(9263\times 10^{-6}\right)$	$\left(2772\times 10^{-7}\right)$	$\left(4749\times 10^{-5}\right)$	$\left(1237\times 10^{-7}\right)$	$\left(1822\times 10^{-5}\right)$
$\beta_{3}$	**0.0183**	0.0189	**0.014**	0.0199	**0.0115**	0.0361	**0.00342**	0.0213
	$\left(3177\times 10^{-5}\right)$	$\left(3499\times 10^{-5}\right)$	$\left(5638\times 10^{-5}\right)$	$\left(8453\times 10^{-5}\right)$	$\left(8302\times 10^{-6}\right)$	$\left(8240\times 10^{-5}\right)$	$\left(1340\times 10^{-6}\right)$	$\left(6036\times 10^{-5}\right)$
$\beta_{4}$	**0.0142**	0.0147	**0.0104**	0.0123	**0.00687**	0.0226	**0.00247**	0.0153
	$\left(1265\times 10^{-5}\right)$	$\left(2337\times 10^{-5}\right)$	$\left(2791\times 10^{-5}\right)$	$\left(3956\times 10^{-5}\right)$	$\left(3368\times 10^{-6}\right)$	$\left(5216\times 10^{-5}\right)$	$\left(4813\times 10^{-7}\right)$	$\left(2507\times 10^{-5}\right)$

**TABLE 4:** Mean and standard deviation (in parentheses) of 100 measures of RMSE when the response follows a Poisson distribution.

Sample size	N=100						N=200					
Domain distribution	Uniform			Negative Binomial			Uniform			Negative Binomial		
Method	BE‐VDFR	VDFR	SOF	BE‐VDFR	VDFR	SOF	BE‐VDFR	VDFR	SOF	BE‐VDFR	VDFR	SOF
$\beta_{1}$	**2.95**	2.96	3.02	**2.76**	2.77	2.91	**2.76**	2,77	2.91	**2.78**	2.79	2.91
	(0.0764)	(0.0760)	(0.1327)	(0.0489)	(0.0493)	(0.1301)	(0.0489)	(0.0493)	(0.1301)	(0.0221)	(0.0216)	(0.1187)
$\beta_{2}$	**2.7**	2.71	2.73	**2.71**	2.71	3.15	**2.71**	2.71	2.69	**2.74**	2.75	2.72
	(0.0453)	(0.0467)	(0.1069)	(0.0445)	(0.0440)	(0.1740)	(0.0208)	(0.0211)	(0.0210)	(0.0169)	(0.0172)	(0.0375)
$\beta_{3}$	**3.62**	3.65	3.72	**7.37**	7.95	9.27	**4.17**	4.22	4.59	**8.58**	9.39	14.7
	(0.2830)	(0.3001)	(0.2432)	(10.5462)	(12.3363)	(16.3661)	(1.2862)	(1.3163)	(5.9494)	(15.6340)	(20.7919)	(25.9246)
$\beta_{4}$	**3.38**	3.41	3.36	**3.77**	3.84	3.72	**3.42**	3.45	3.41	**4.11**	4.26	4.12
	(0.3944)	(0.4294)	(0.9920)	(0.2435)	(0.2611)	(0.3225)	(0.1979)	(0.2136)	(0.4749)	(0.4118)	(0.4989)	(1.0965)

The results demonstrate that the BE‐VDFR model consistently outperforms other methodologies across all scenarios. Specifically, for AMSE, the BE‐VDFR model shows lower average errors than the VDFR model across all simulated data sets, with differences reaching up to an order of magnitude in some cases.

Regarding RMSE, the BE‐VDFR model again achieves superior performance, displaying the lowest mean error in nearly all scenarios. The only exception occurs in a scenario with true functional coefficient $\beta_{3}$, a Normal response distribution, a sample size of N=200, and a uniform domain distribution, where the SOF model has a slightly lower mean error. However, the RMSE variability in this scenario indicates greater stability for the BE‐VDFR model, with the lowest variability at 2.09 compared to 5.42 and 4.15 for the VDFR and SOF methods, respectively.

**FIGURE 2:**
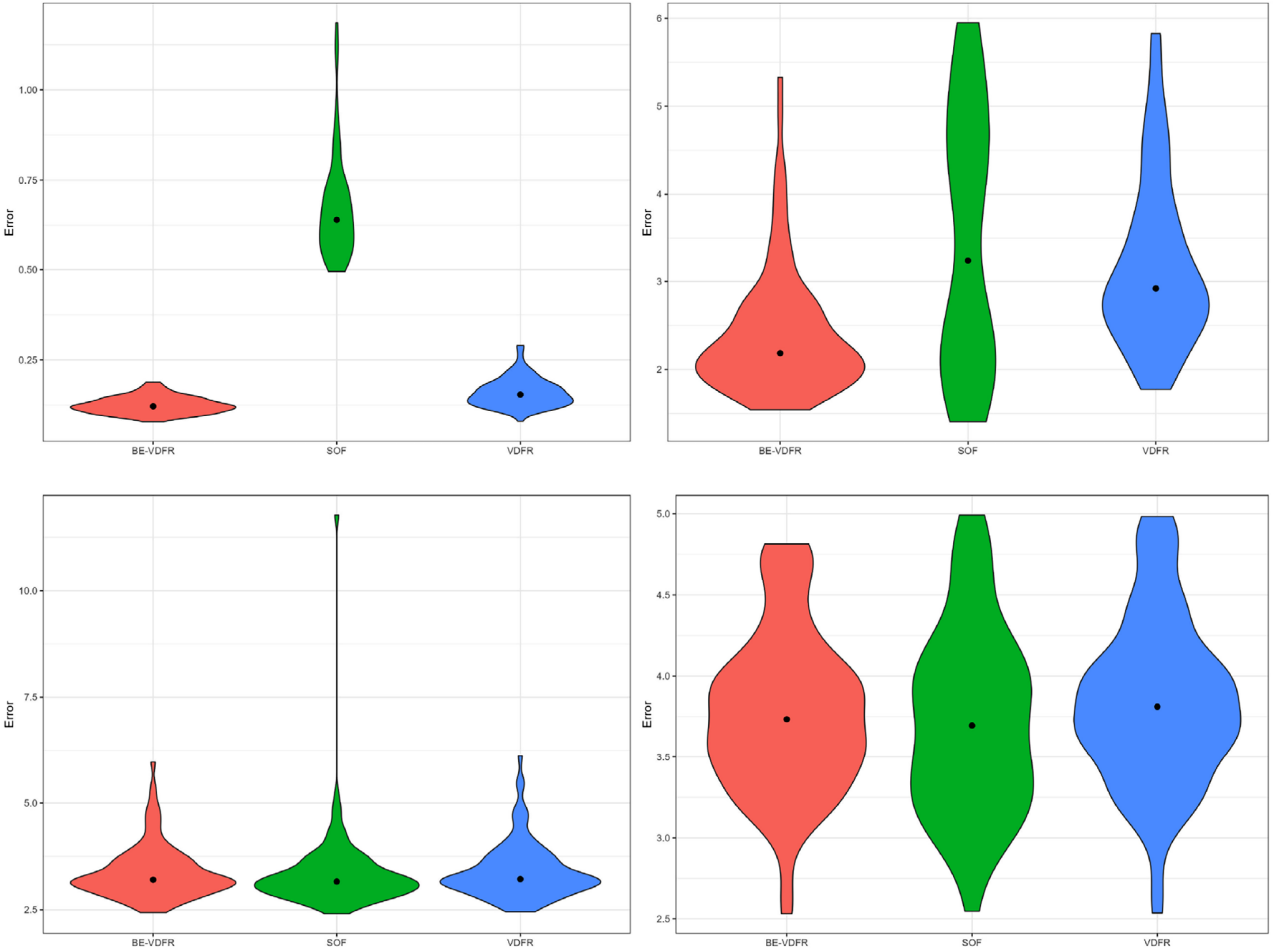
Violin box‐plots of the RMSE for scenarios where the true functional coefficient is $\beta_{4}\left(t,T\right)$ and the sample size is N=100. The top row corresponds with the response variable following a Normal distribution while the bottom row shows its Poisson counterpart. The left column corresponds to the domain following a Uniform distribution and the right column represents the domain following a Negative Binomial distribution. The methods are from left to right: BE‐VDFR, SOF, and VDFR.

**FIGURE 3:**
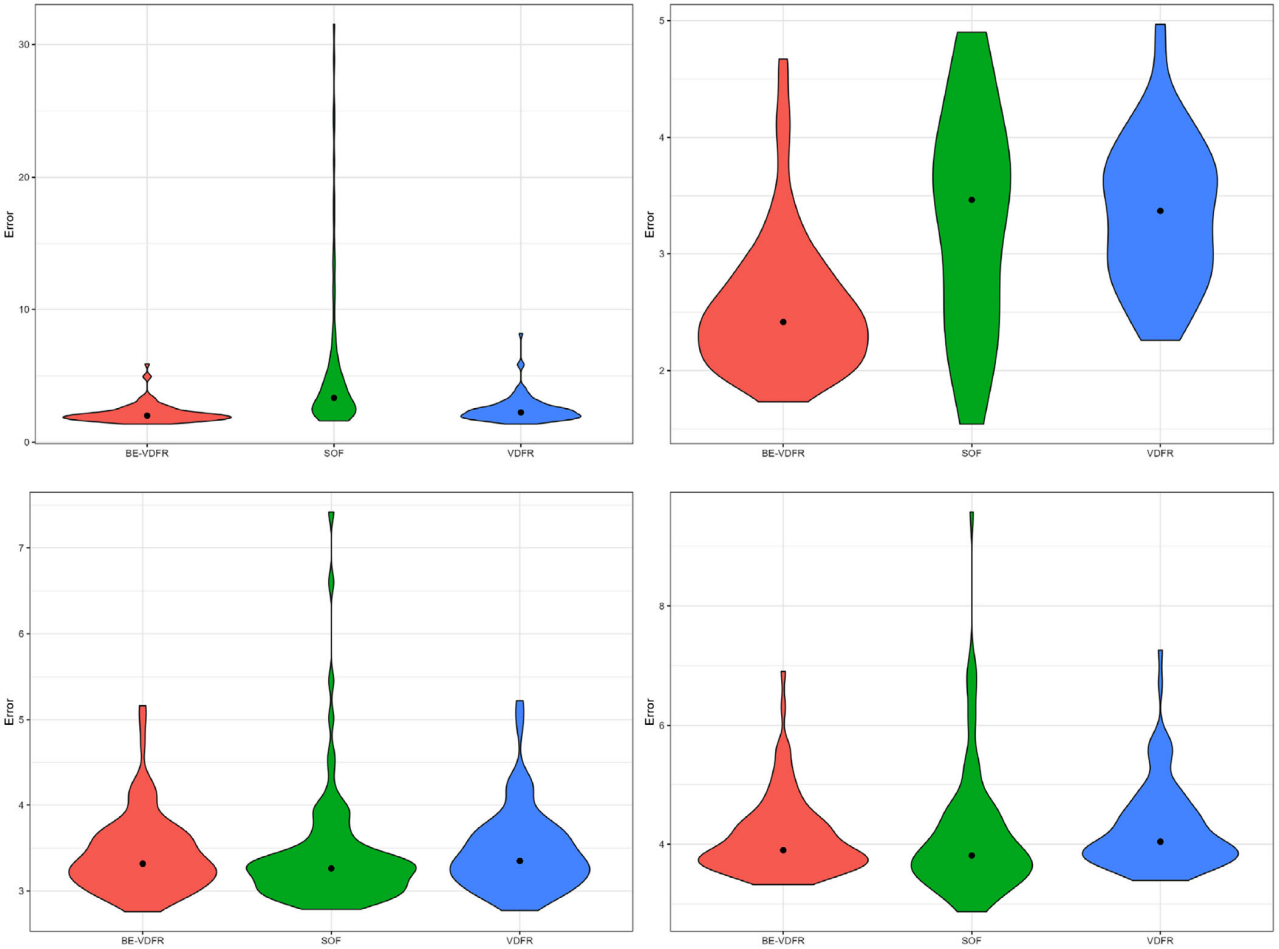
Violin box‐plots of the RMSE for scenarios where the true functional coefficient is $\beta_{4}\left(t,T\right)$ and the sample size is N=200. The top row corresponds with the response variable following a Normal distribution while the bottom row shows its Poisson counterpart. The left column corresponds to the domain following a Uniform distribution and the right column represents the domain following a Negative Binomial distribution. The methods are from left to right: BE‐VDFR, SOF, and VDFR.

**FIGURE 4:**
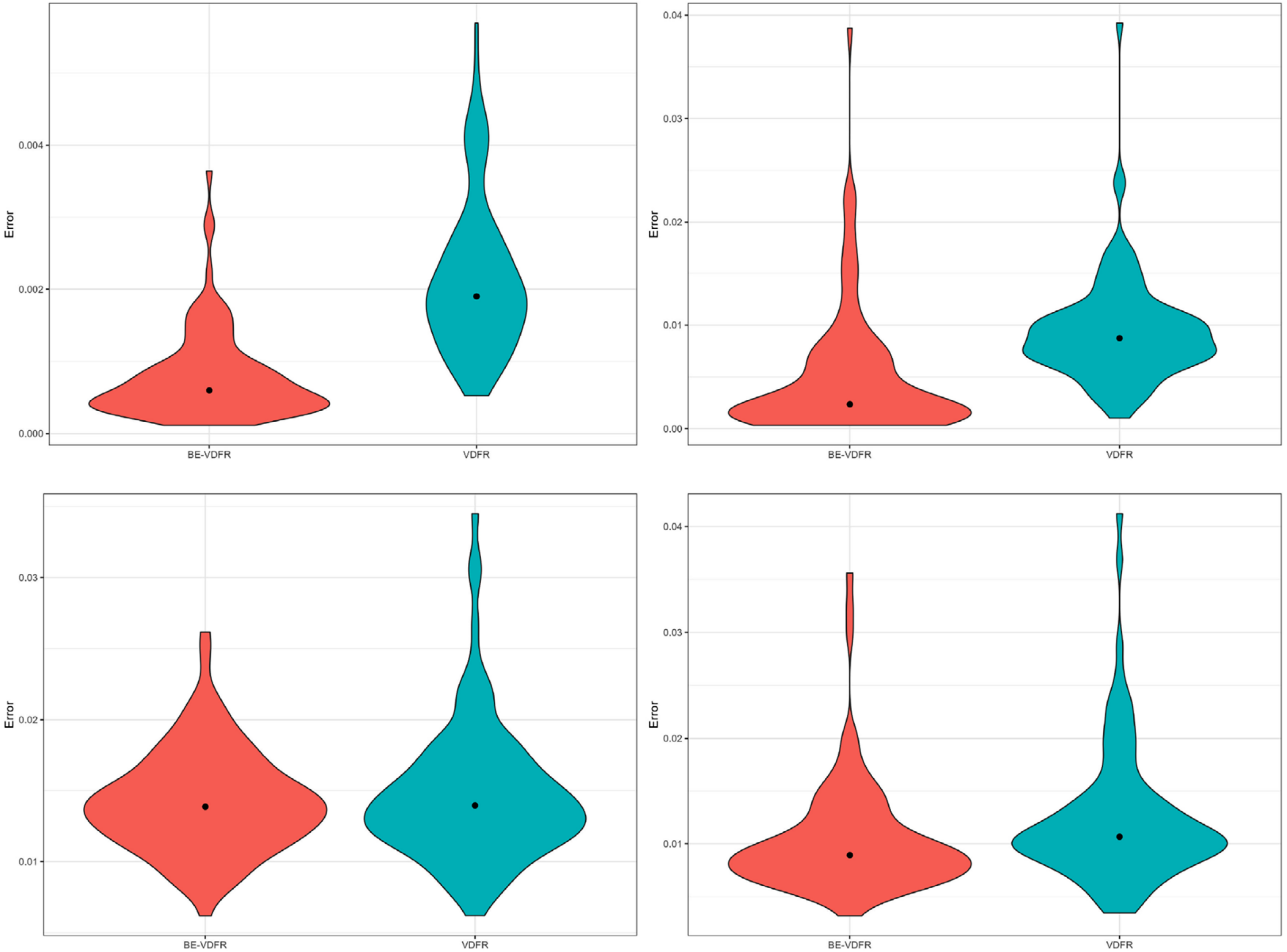
Violin box‐plots of the AMSE for scenarios where the true functional coefficient is $\beta_{4}\left(t,T\right)$ and the sample size is N=100. The top row corresponds with the response variable following a Normal distribution while the bottom row shows its Poisson counterpart. The left column corresponds to the domain following a Uniform distribution and the right column represents the domain following a Negative Binomial distribution. The methods are from left to right: BE‐VDFR and VDFR.

**FIGURE 5:**
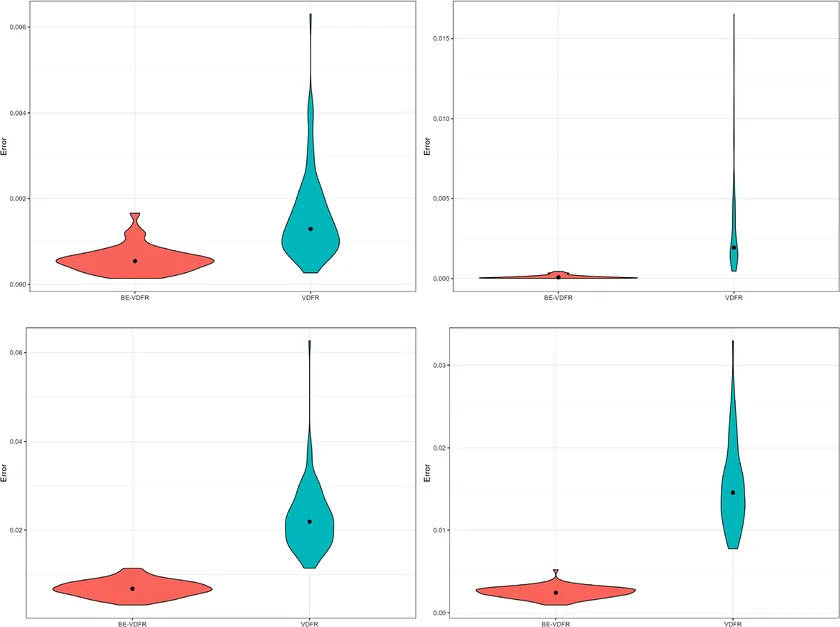
Violin box‐plots of the AMSE for scenarios where the true functional coefficient is $\beta_{4}\left(t,T\right)$ and the sample size is N=200. The top row corresponds with the response variable following a Normal distribution while the bottom row shows its Poisson counterpart. The left column corresponds to the domain following a Uniform distribution and the right column represents the domain following a Negative Binomial distribution. The methods are from left to right: BE‐VDFR and VDFR.

In addition to the tables summarizing the simulation study, Figures 2, 3, 4, 5 present the detailed visualizations of the error variability across multiple scenarios using violin plots. These plots allow for a comprehensive comparison between methodologies, highlighting the performance of each approach under various conditions. As we will describe next, the superior performance of the BE‐VDFR method is consistently demonstrated across all metrics and performance criteria.

Figure 2 illustrates the RMSE distribution for scenarios with a sample size of N=100. The BE‐VDFR method exhibits consistently lower error variability across all scenarios. Its advantage is particularly evident when the response follows a Normal distribution, as the variability in the errors is lower, and the maximum error threshold is consistently below the highest errors produced by the VDFR and SOF methods. In contrast, the SOF method demonstrates the highest variability in its error distribution, with wider violin plots and more extreme maximum error values. When the sample size is to N=200, as shown in Figure 3, the distinctions between methodologies become more pronounced. The BE‐VDFR method maintains its robust performance with compact and well‐contained error distributions, while the SOF method exhibits even greater variability in its error distribution. Furthermore, in scenarios where the domain follows a Negative Binomial distribution, the BE‐VDFR method achieves significantly lower median errors and maintains its consistent advantage over other methodologies.

Turning to the AMSE criterion, the advantages of the BE‐VDFR method become even more apparent. In Figure 4 (corresponding to a sample size of N=100), BE‐VDFR method consistently shows reduced error variability across all scenarios. This improvement is particularly evident when the domain follows a Uniform distribution, where both the spread and maximum error values for the BE‐VDFR method are notably smaller compared to the VDFR method. The median AMSE values further confirm the superior performance of the BE‐VDFR method, especially in scenarios where the response follows a Normal distribution.

In Figure 5, where the sample size increases to N=200, the advantages of the BE‐VDFR method are even more pronounced. The violin plots show that the error distributions for the BE‐VDFR method are tightly clustered, reflecting a significant reduction in variability. Maximum error values are substantially lower than those for the VDFR method, and the median errors remain consistently smaller across all scenarios. the superior performance of the BE‐VDFR method is evident across various sample sizes, response distributions, and domain scenarios.

**FIGURE 6:**
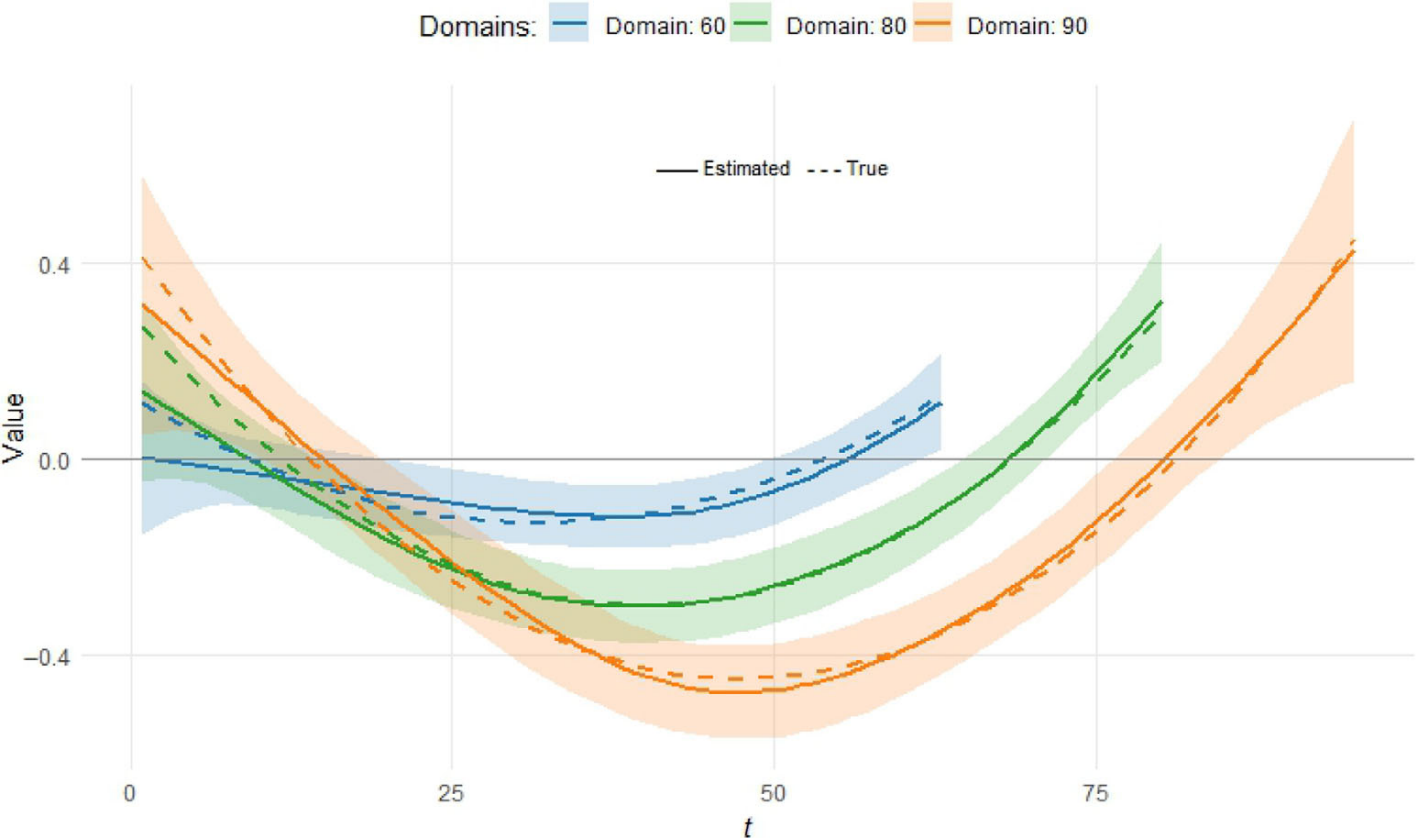
Confidence intervals for the case where the true functional coefficient is $\beta_{2}\left(t,T\right)$, the response variable follows a normal distribution, and the domain follows a Uniform distribution. The solid and dashed lines represent the estimated and true functional coefficients, respectively. The shaded area represents the 95% confidence interval.

In addition to the previous description of the simulation study results, the confidence bands for the estimation of the functional coefficient were calculated for the proposed methodology following the procedure shown in the previous section. Some confidence intervals corresponding to specific domain values are shown in Figure 6. Here, it can be seen how, almost everywhere in the curve, the confidence interval contains the true value of the corresponding curve of the real functional coefficient. In addition, the confidence interval holds the sign of the estimated functional coefficient, that is, when the estimation is positive/negative the confidence interval stays within positive/negative values, meaning that the estimation is statistically significant.

**FIGURE 7:**
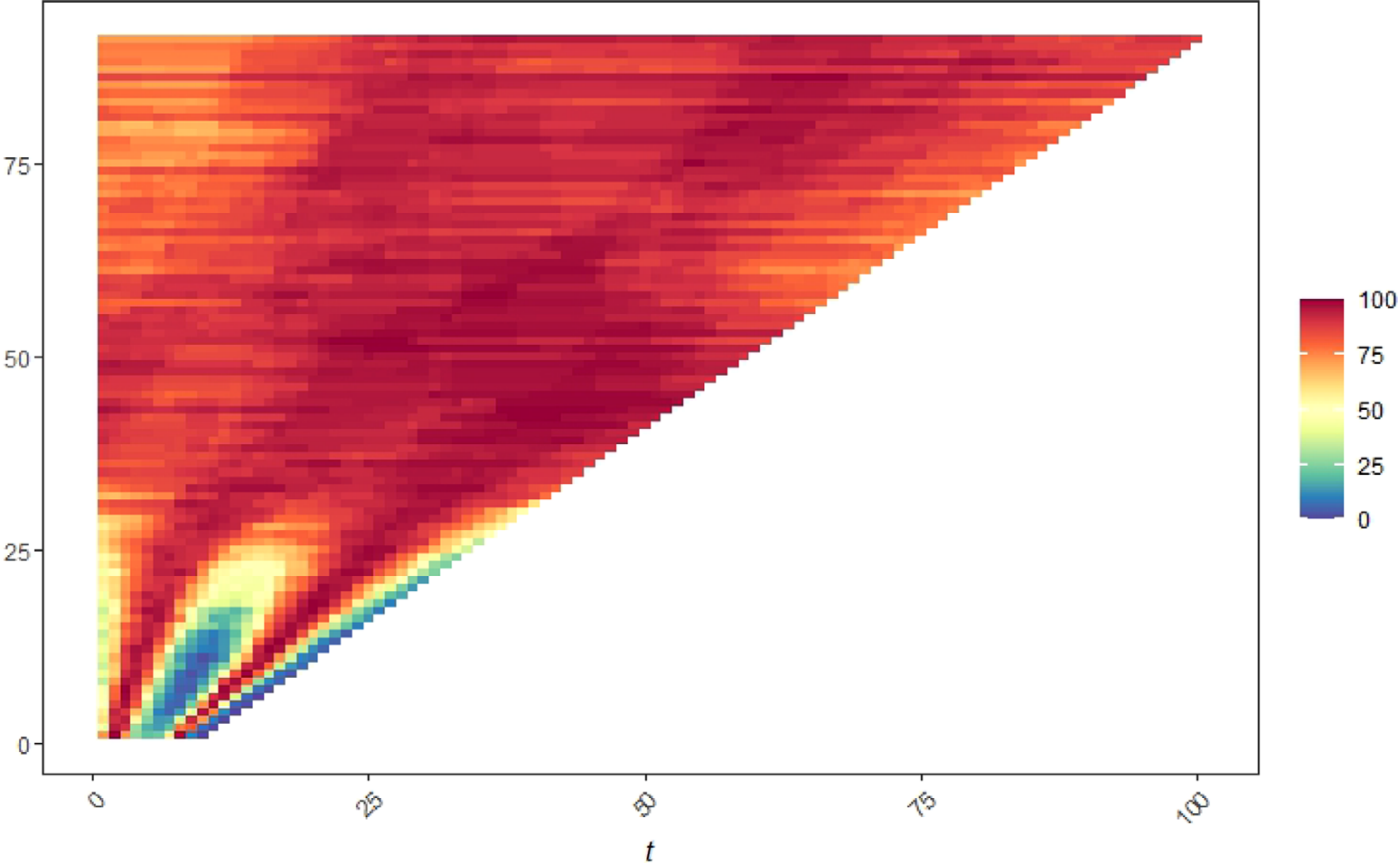
Coverage for the case where the true functional coefficient is $\beta_{2}\left(t,T\right)$ the response variable follows a Poisson distribution and the domain follows a Uniform distribution.

To further analyze the consistency of the proposed methodology, a more detailed study concerning the confidence interval has been carried out and it is shown in Figure 7. This figure shows a heat map with values of the coverage (in percentage) for every point of the true functional coefficient for the scenario corresponding with the response variable following a Poisson distribution, the domain following a Uniform distribution and the true functional coefficient being $\beta_{2}\left(t,T\right)$.

Notice that for different repetitions of the simulation, the functional coefficient is not the same and does not have the same domain for all its curves across repetitions. For example, the shorter curve of the first repetition of the simulation has a domain T=10, while for the second repetition, the shorter domain is T=12. To solve this, all the curves with the same domain across all repetitions have been considered to calculate the coverage. Following this idea, the coverage for the first point of one curve with a specific domain is the number of times that the first point of all curves with the same domain falls within its corresponding confidence interval. This procedure is applied to all points across all curves and domains. This provides an overall idea of the behavior of the proposed methodology in terms of coverage of the true functional coefficient. In addition, as a summarizing statistic for this particular scenario, it can be found that the median coverage is 94.4%.

Figure 7 predominantly shows warm colors, with a notable presence of red, indicating a coverage value close to 100%. This means that the confidence intervals calculated using the proposed methodology cover the true functional coefficient almost all the time across all the different simulations. Regions with cooler colors are observed in areas where the domain is shorter (T<20). The lower performance in areas where the domain is relatively shorter could be expected since the number of knots of the B‐spline basis corresponding to the curves in that area is proportional to the total number of knots, resulting in a smoother estimation curve. Nonetheless, this limitation does not undermine the methodology, as increasing the number of basis functions can effectively address this issue, ensuring reliable performance across all domains.

## 5 Case Study: The telEPOC Data Set

The telEPOC program (Esteban et al. 2016) was conducted at the Galdakao‐Usansolo University Hospital (Vizcaya, Spain). Patient recruitment took place from 2010 to 2013, with a follow‐up period of 5 years. The primary aim of the study was to evaluate the effectiveness of a telemonitoring program (telEPOC) for COPD patients with frequent hospitalizations. A total of 119 patients, identified as those with a history of frequent hospitalizations, were selected for home telemonitoring.

Moreover, one of the goals of the study was to analyze the effect of performing physical activity on the health of the patients, in particular on the rate of hospitalizations due to COPD. The performance of daily physical activity was measured as the number of daily steps taken by each patient during their time in the study, which was included in the telemonitoring process. This has been the motivation of the work we present in this paper.

Patients were included in the study at different time points. However, we are not interested in the effect, if any, that the different dates of admission on patients may have. For that reason, and a clearer analysis, we have aligned to the left all the collected variables making all patients begin the study at “day 1.”

Of the initial 119 patients, only 112 had complete step count records. Two additional patients with irregular measurements were excluded. Furthermore, measurements outside a realistic range of daily steps were treated as missing data and were imputed by averaging adjacent days. Consequently, our final data set consisted of 110 patients with complete daily physical activity records, highlighting the importance of data smoothing for reliable results.

The study also collected clinical variables at baseline as possible covariates of interest: smoking habits, age, gender, previous hospitalizations due to COPD, anxiety, and depression symptomatology, among others. The number of hospitalizations due to COPD during the time in the study, the mortality, and the time spent in the study were recorded as potential outcomes. For a detailed explanation of the data collected in the study as well as the enrollment procedures and criteria of acceptance, we refer the readers to Esteban et al. (2016).

The response variable in this analysis is the annual rate of hospitalizations, with daily physical activity (measured in steps) as the main covariate. To account for the varying lengths of follow‐up, we used the annual rate of hospitalizations, instead of the number hospital admissions, mitigating any cumulative time effects. Then, BE‐VDFR and VDFR with Poisson response were used to analyze the data. The BE‐VDFR considered with B‐spline bases of 25 for the functional covariate, and 625 for the bidimensional coefficient (25 for each marginal basis), while in the case of VDFR the functional coefficient was represented by thin‐plate spline bases basis (as suggested in Gellar et al. 2014), a total of 1200 basis were used (the máximun allowed by the software). Parameter estimation was carried out using PQL, the Akaike Information criterion (AIC, Akaike 1974) was used for variable selection and model comparison.

### 5.1 Results

The AIC criterion identified four baseline covariates: gender, prior hospitalizations, anxiety, and depressive symptomatology. As a result, the final model incorporates one functional covariate along with these four baseline variables. The estimated coefficients (see Table 5) indicate that female gender, a history of hospitalizations, and depressive symptomatology are each associated with increased hospitalization rates, whereas anxiety shows a potentially protective effect. This combination of effects implies that patients who are female, have been previously hospitalized, and present depressive symptoms—but do not exhibit anxiety—may have a higher risk profile. The exclusion of zero from all confidence intervals supports the statistical significance of these associations

**TABLE 5:** Estimated coefficients with 95% confidence intervals shown in parentheses of the four baseline covariates.

Variable	Coefficient (95% CI)
Gender (female)	0.848 (0.270, 1.426)
Previous COPD hospitalizations	0.085 (0.019, 0.152)
Anxiety	−0.106 (−0.169, −0.043)
Depression	0.114 (0.046, 0.182)

Nevertheless, for the interpretation of the results, we will focus on the effect of the functional variable on the annual rate of hospitalizations, via the functional coefficient $\hat{\beta }\left(t,T\right)$, which reflects directly the relationship between the daily number of steps and the annual rate of hospitalizations due to COPD, adjusted by the baseline covariates in the model.

A negative value of the functional coefficient $\hat{\beta }\left(t,T\right)$ suggests a beneficial impact on the patient's health, indicating that physical activity contributes to a reduction in the annual hospitalization rate. Conversely, a positive value suggests that physical activity might be associated with an increase in hospitalization rates. However, these interpretations should be approached cautiously, as no conclusive evidence establishes a significant effect in either direction.

The estimated functional coefficient is represented as a surface. For a fixed value of $T=T_{i}$, the resulting curve illustrates the influence of physical activity on the annual hospitalization rate for patients who engaged in physical activity for $T_{i}$ days.

**FIGURE 8:**
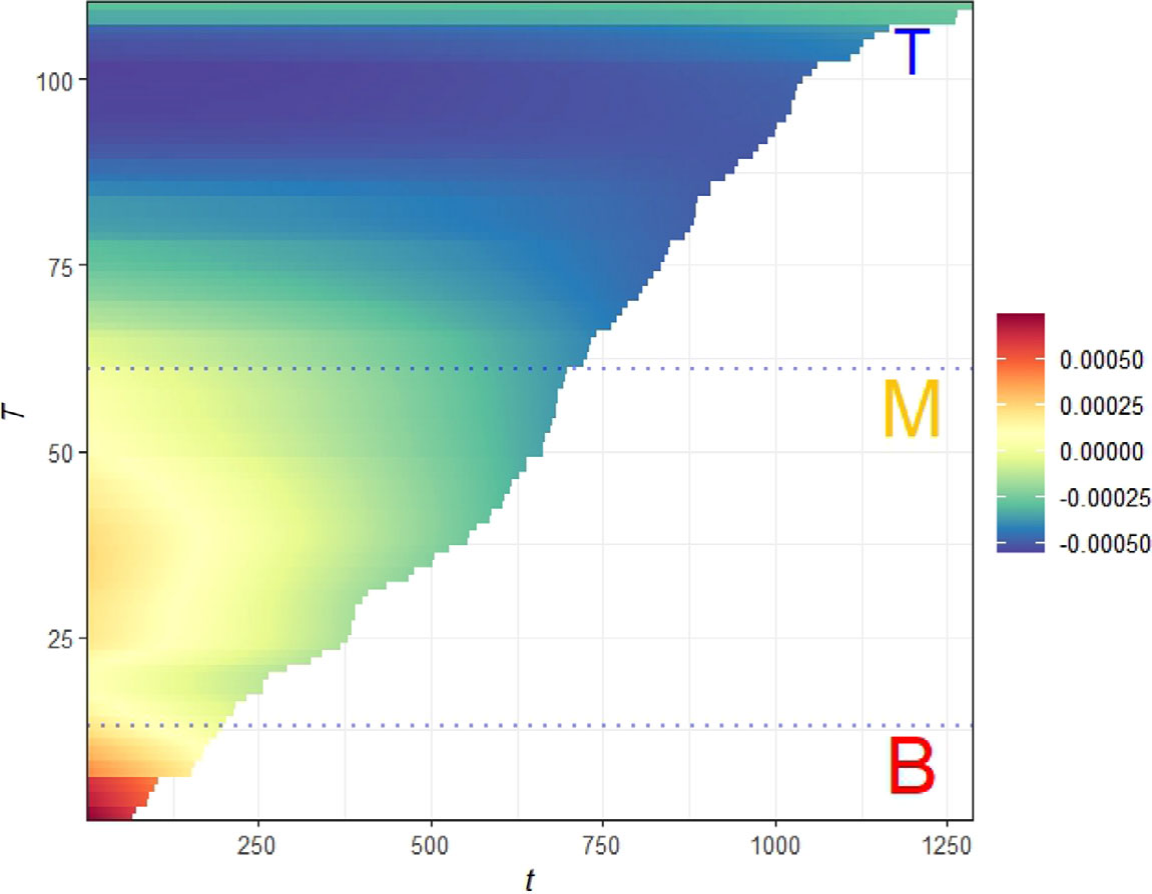
Functional coefficient $\beta (t,T_{i})$ for patients with $T_{i}$ days in the study.

Figure 8 presents a heat map of the estimated surface, $\hat{\beta }\left(t,T\right)$, for all periods in which patients engaged in physical activity. A common trend emerges from the heat map: regular physical activity helps reduce the average annual hospitalization rate due to COPD. This is evident because the curves either decrease or exhibit negative values, represented in the heat map by cool colors (green or blue), signifying a favorable influence of physical activity on reducing hospitalizations.

For patients whose corresponding curves are shorter than 194 days (approximately 6 months), represented at the bottom of the heat map and marked with the red letter **B**, the curves often end in warm colors (red or orange), indicating positive values. This suggests that engaging in physical activity for less than 6 months is insufficient to reduce the annual hospitalization rate. However, the downward trend of these curves still indicates that physical activity positively impacts the health of these patients.

A closer examination of the heat map reveals patterns of similarity among patients. Three distinct regions can be identified:

1. **Bottom Region (marked with the red letter B):** This area represents patients who engaged in physical activity for less than 6 months. While their curves end with positive values, signifying insufficient time for a substantial reduction in hospitalizations, the decreasing trend shows a gradual improvement in health.
2. **Middle Region (marked with the yellow letter M):** This region corresponds to patients who performed physical activity for between 194 days (6 months) and 696 days (approximately 2 years). These curves typically start with positive values but become negative and remain so, indicating a clear reduction in the annual hospitalization rate over time.
3. **Top Region (marked with the blue letter T):** In this area, the curves are negative throughout their entire domain, indicating that patients who performed physical activity for over 2 years experienced consistent reductions in their annual hospitalization rates, demonstrating significant health improvements.

In conclusion, the analysis indicates that physical activity has a positive impact on reducing the annual hospitalization rate due to COPD within this patient group. Specifically, patients who engage in physical activity for at least 6 months are likely to see a reduction in their hospitalization rates. Longer durations of physical activity yield progressively greater benefits.

A potential limitation of our study is the lack of consideration for the longitudinal effects of other COPD‐related variables—such as disease progression or deterioration—on patient outcomes. Although the main predictors of health‐related quality of life evolution were included in the analysis (Esteban et al. 2020), a more comprehensive examination could yield additional insights.

## 6 Discussion

In this paper, we introduced a novel model to analyze variable domain functional data. The proposed approach is based on the basis representation of both the functional data and the functional coefficient, transforming the original functional model into a multivariate one. This multivariate formulation is subsequently reparameterized as a mixed model to gain computational efficiency. Referred to as BE‐VDFR model, our approach addresses key limitations of existing methodologies, particularly by enabling the optimal use of anisotropic penalties. Although certain variants of the original VDFR model can potentially address anisotropy, a key advantage of our approach lies in its flexibility to accommodate both isotropic and anisotropic scenarios within a unified framework.

We evaluated the performance of the BE‐VDFR model through an extensive simulation study, comparing it against both the standard SOF model and the VDFR model based on TPRS proposed by Gellar et al. (2014). The results consistently showed that the BE‐VDFR model outperformed these alternatives across all considered evaluation criteria, highlighting its superior accuracy and robustness.

The method was developed with the specific goal of assessing the impact of physical activity on the annual rate of hospitalizations in COPD patients, using data from the telEPOC program. Our findings revealed that maintaining regular physical activity for at least 6 months leads to a significant reduction of the annual rate of hospitalizations due to COPD. This highlights the critical role of sustained physical activity in managing COPD‐related health outcomes.

While prior research has documented an association between physical activity and hospitalization risk in COPD patients (Garcia‐Rio et al. 2012), to the best of our knowledge, no previous study has tracked daily physical activity at the resolution required to establish a detailed relationship with hospitalization outcomes. The telEPOC program provides valuable daily step count data that not only assists clinicians in monitoring patients and identifying potential exacerbations, but also supports midterm strategies to enhance patients' health‐related quality of life (Esteban et al. 2020).

A current limitation of the proposed methodology lies in its treatment of uncertainty: specifically, the estimated basis coefficients ${\hat{a}}_{i}$ are treated as fixed when computing confidence intervals for $\hat{\beta }\left(t,T\right)$, following standard practice in two‐step functional data analysis (Aguilera‐Morillo et al. 2013). While this approach is computationally efficient, it may underestimate the true variability by not propagating uncertainty from the curve reconstruction step. A fully Bayesian hierarchical model could address this limitation by jointly modeling both the basis coefficients and the functional regression parameters, albeit at considerably greater computational cost.

Future research should examine the practical impact of this simplification and seek to develop efficient strategies for comprehensive uncertainty quantification in VDFR. Another promising direction for future work is to extend the BE‐VDFR framework to function‐on‐function regression models in which either the regressors, the response variable, or both, vary over observation‐dependent domains.

In conclusion, our proposal effectively incorporates the variable domain feature into a functional data analysis framework, offering a robust solution for analyzing data with observation‐dependent domains.

Finally, the BE‐VDFR model has been implemented in an R package, **VDPO**, which is publicly available on CRAN and can be explored in more detail through its https://pavel‐hernadez‐amaro.github.io/VDPO/.

## Conflicts of Interest

The authors declare no conflicts of interest.

## Open Research Badges

This article has earned an Open Data badge for making publicly available the digitally‐shareable data necessary to reproduce the reported results. The data is available in the Supporting Information section.

This article has earned an open data badge “**Reproducible Research**” for making publicly available the code necessary to reproduce the reported results. The results reported in this article could fully be reproduced.

## Supporting information


**Supporting File:** bimj70164‐sup‐0001‐SuppMat.pdf.

## Data Availability

The data that support the findings of this study are openly available in Simulation Results at https://doi.org/10.5281/zenodo.15741032.
