# Supplementary material for: Modeling Physical Activity Profiles in COPD Patients: A Basis Expansion Approach to Variable Domain Functional Regression Models
Source: Biom J. 2026 Aug 27;68(5):e70164. doi: 10.1002/bimj.70164 (PMC13520829; doi:10.1002/bimj.70164)
Supplement: Supplementary file 1 [file BIMJ-68-e70164-s001.pdf]

Biometrical Journal (2025) , zzz–zzz / DOI: 10.1002/

Modelling physical activity profiles in COPD patients: a basis expansion approach to variable domain functional regression models: Supplementary Material

Pavel Hernández-Amaro[*,1], **María Durbán** [1], **M. Carmen Aguilera-Morillo** [2], **Cristobal Esteban Gonzalez** [3], and **Inmaculada Arostegui** [4]

[1] Universidad Carlos III de Madrid

[2] Universitat Politècnica de València

[3] Osakidetza Basque Health Service

[4] University of the Basque Country UPV/EHU

This document contains supplementary material for the paper ”Modelling physical activity profiles in COPD patients: a basis expansion approach to variable domain functional regression models”. The supplementary material includes additional simulation results with detailed violin plots for all scenarios evaluated in the simulation study, providing comprehensive visual evidence of the superior performance of the BE-VDFR method compared to existing approaches.

1 Additional Simulation Results

This section presents comprehensive simulation results through violin plots for all evaluated scenarios. The figures show detailed comparisons between the BE-VDFR method, VDFR method, and SOF method across different functional coefficient configurations (β_1 through β_4), sample sizes ($N = 100, 200$), response distributions (Normal and Poisson), and domain distributions (Uniform and Negative Binomial).

Each violin plot displays the distribution of errors across 100 simulation iterations, allowing for a detailed assessment of both central tendency and variability of the performance measures. The plots consistently demonstrate the superior performance and stability of the BE-VDFR method across most of the considered scenarios.

1.1 RMSE Results for N=100

Figure 1 through Figure 4 present the Root Mean Square Error (RMSE) distributions for scenarios with sample size $N = 100$ across all four functional coefficient configurations.

*Corresponding author: e-mail: pahernan@est-econ.uc3m.es

© 2025 WILEY-VCH Verlag GmbH & Co. KGaA, Weinheim

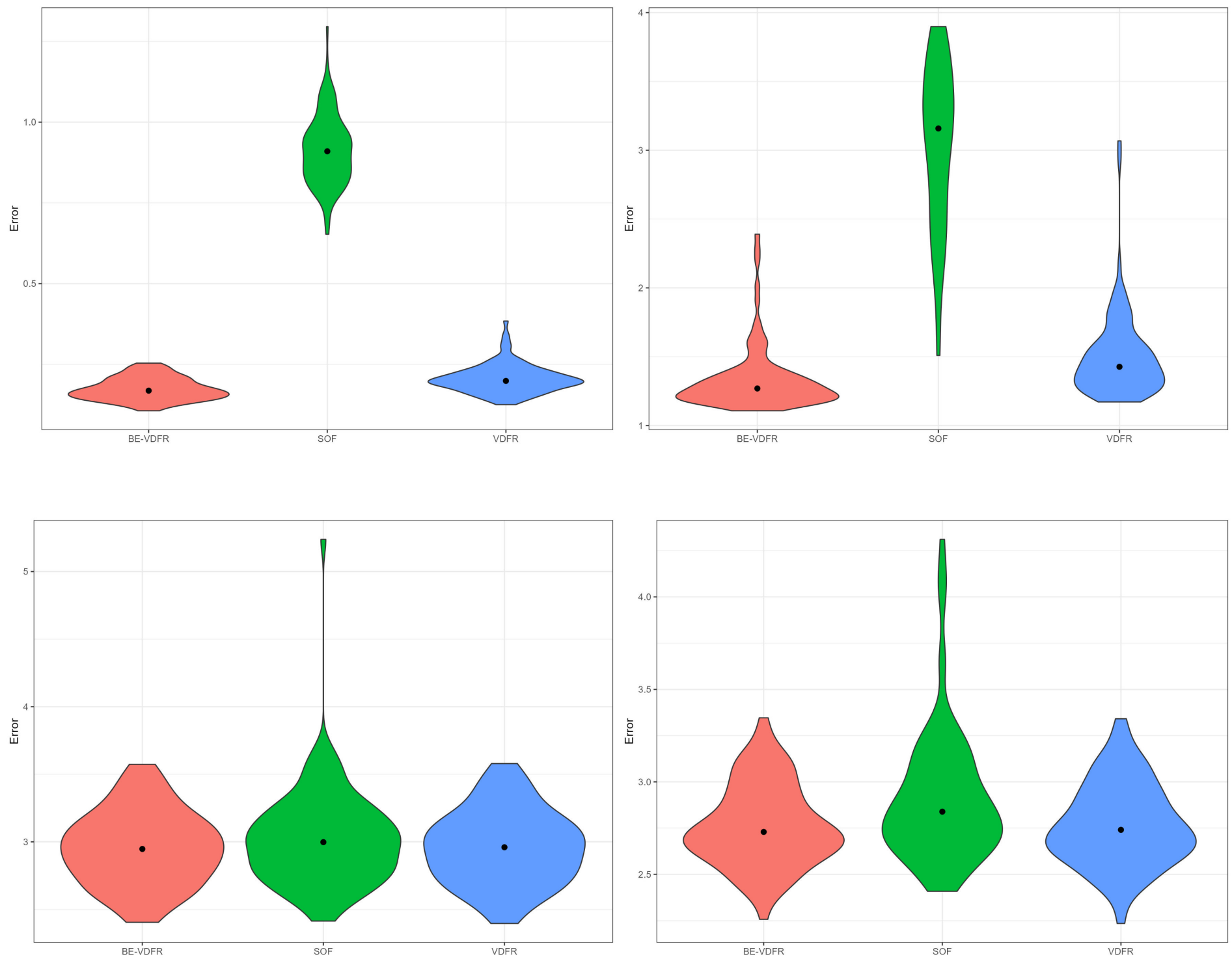

Figure 1 Violin box-plots of the RMSE for scenarios where the true functional coefficient is $\beta_1(t,T)$ and the sample size is $N = 100$. The top row corresponds with the response variable following a Normal distribution while the bottom row shows its Poisson counterpart. The left column corresponds to the domain following a Uniform distribution and the right column represents the domain following a Negative Binomial distribution. The methods are from left to right: BE-VDFR, SOF, VDFR.

© 2025 WILEY-VCH Verlag GmbH & Co. KGaA, Weinheim

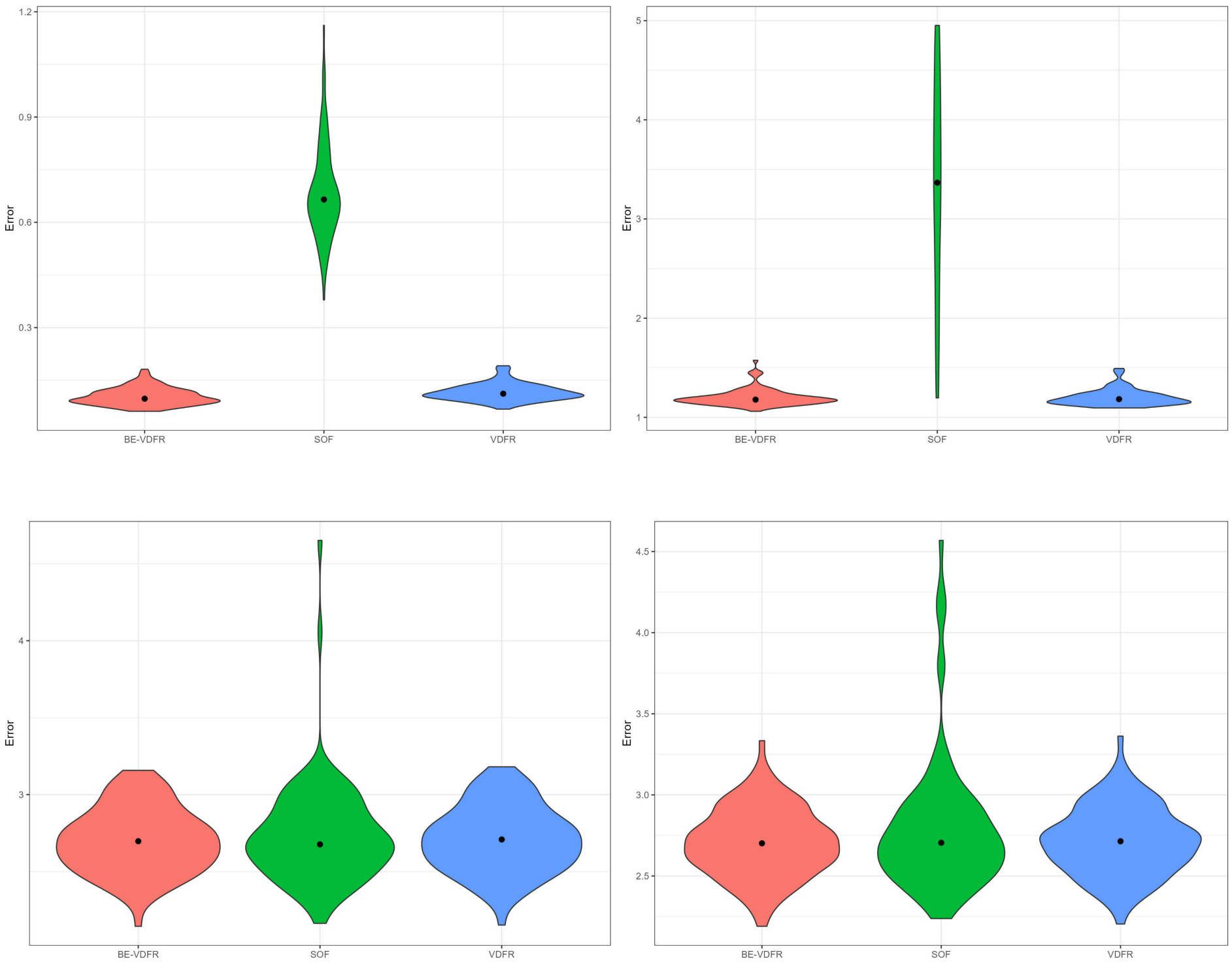

Figure 2 Violin box-plots of the RMSE for scenarios where the true functional coefficient is $\beta_2(t, T)$ and the sample size is $N = 100$. The top row corresponds with the response variable following a Normal distribution while the bottom row shows its Poisson counterpart. The left column corresponds to the domain following a Uniform distribution and the right column represents the domain following a Negative Binomial distribution. The methods are from left to right: BE-VDFR, SOF, VDFR.

© 2025 WILEY-VCH Verlag GmbH & Co. KGaA, Weinheim

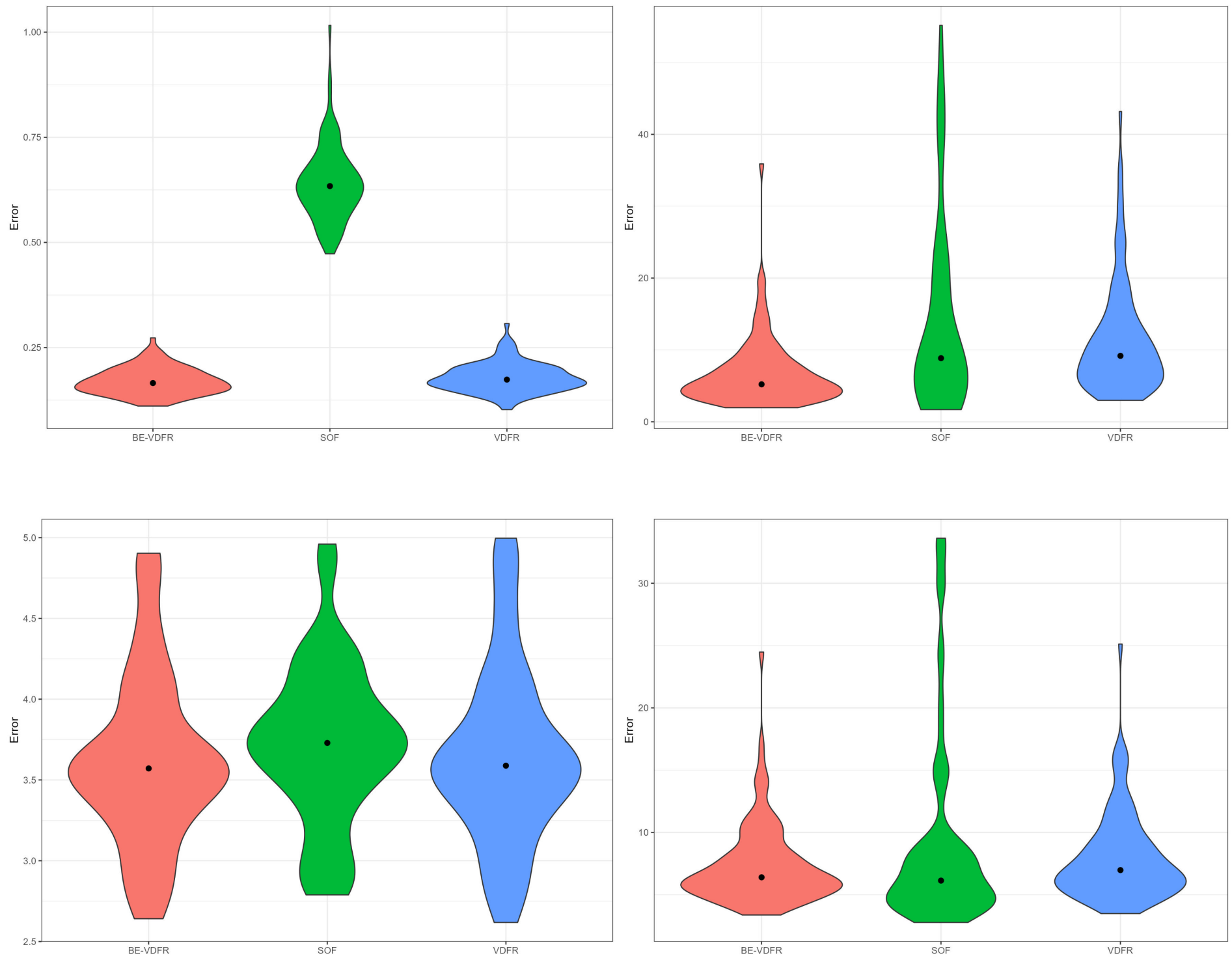

Figure 3 Violin box-plots of the RMSE for scenarios where the true functional coefficient is $\beta_3(t, T)$ and the sample size is $N = 100$. The top row corresponds with the response variable following a Normal distribution while the bottom row shows its Poisson counterpart. The left column corresponds to the domain following a Uniform distribution and the right column represents the domain following a Negative Binomial distribution. The methods are from left to right: BE-VDFR, SOF, VDFR.

© 2025 WILEY-VCH Verlag GmbH & Co. KGaA, Weinheim

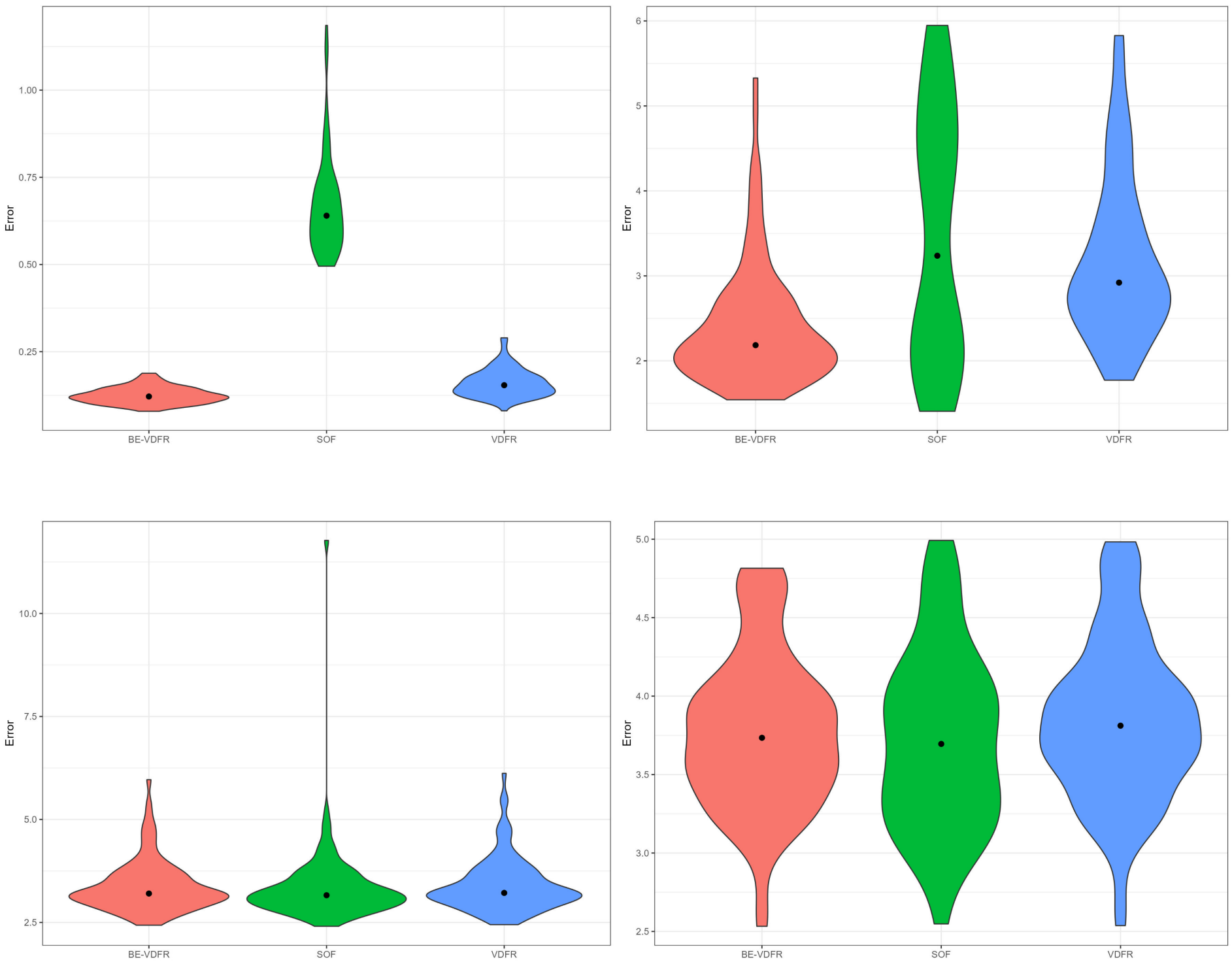

Figure 4 Violin box-plots of the RMSE for scenarios where the true functional coefficient is $\beta_4(t, T)$ and the sample size is $N = 100$. The top row corresponds with the response variable following a Normal distribution while the bottom row shows its Poisson counterpart. The left column corresponds to the domain following a Uniform distribution and the right column represents the domain following a Negative Binomial distribution. The methods are from left to right: BE-VDFR, SOF, VDFR.

© 2025 WILEY-VCH Verlag GmbH & Co. KGaA, Weinheim

1.2 RMSE Results for N=200

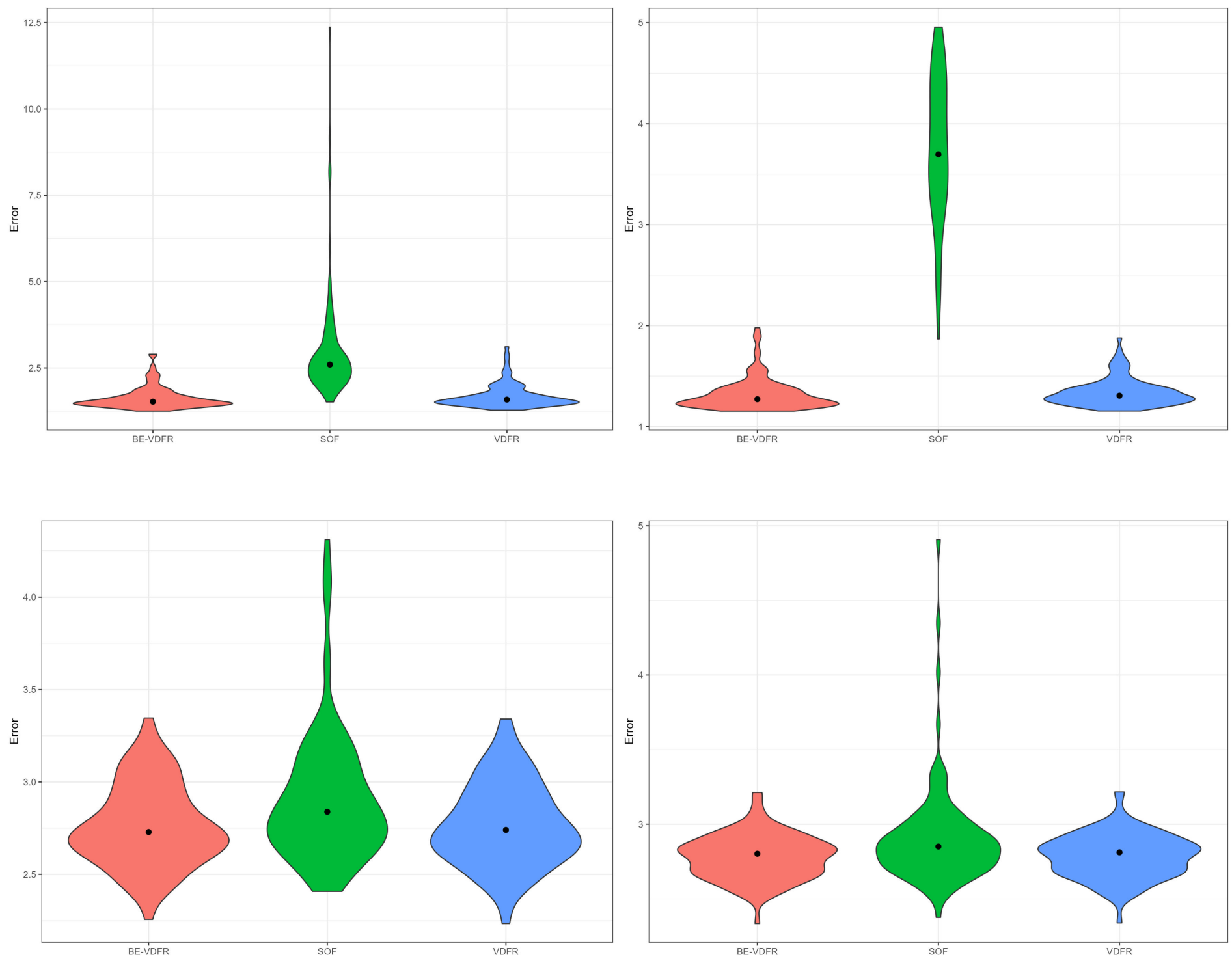

Figure 5 Violin box-plots of the RMSE for scenarios where the true functional coefficient is $\beta_1(t,T)$ and the sample size is $N = 200$. The top row corresponds with the response variable following a Normal distribution while the bottom row shows its Poisson counterpart. The left column corresponds to the domain following a Uniform distribution and the right column represents the domain following a Negative Binomial distribution. The methods are from left to right: BE-VDFR, SOF, VDFR.

© 2025 WILEY-VCH Verlag GmbH & Co. KGaA, Weinheim

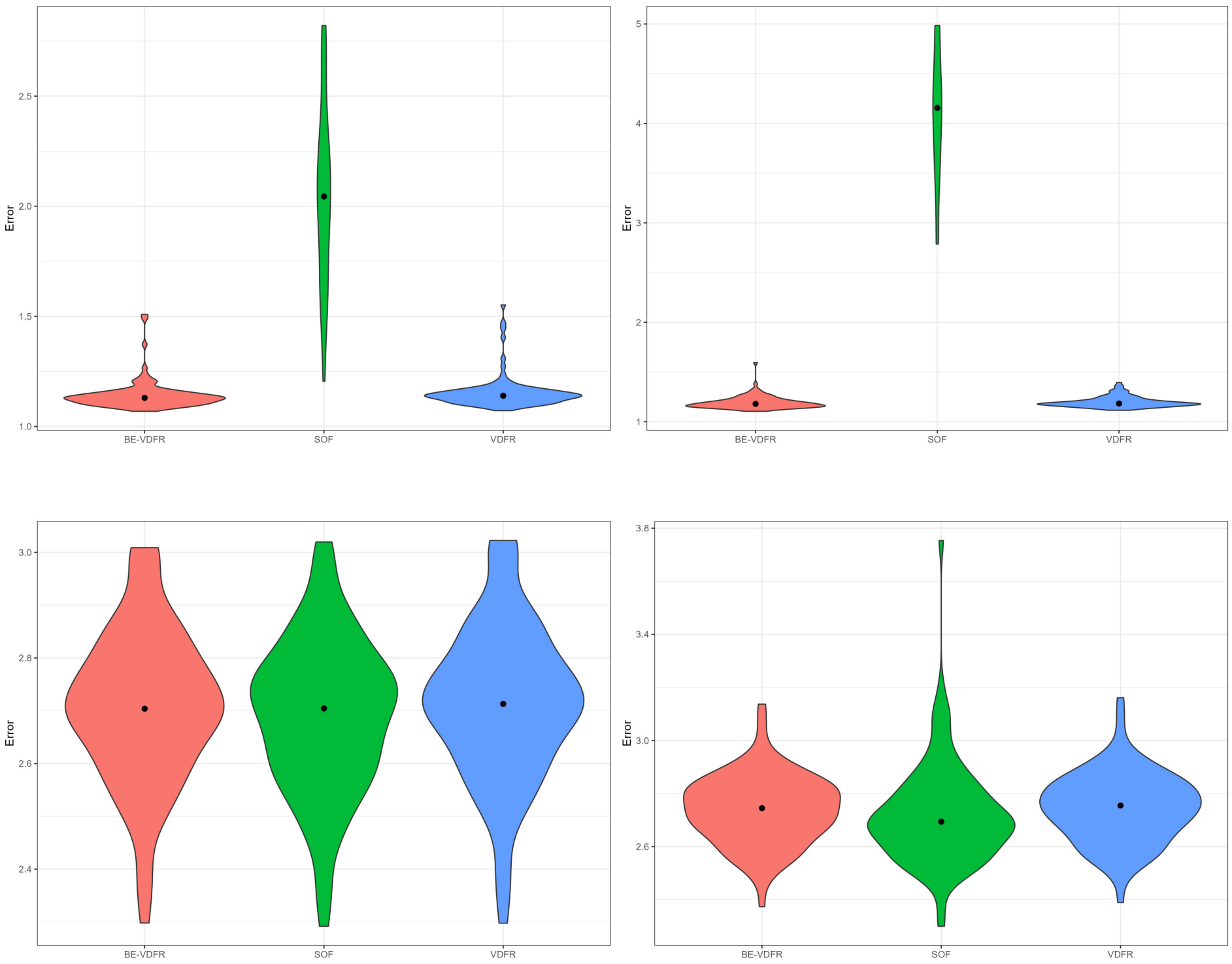

Figure 6 Violin box-plots of the RMSE for scenarios where the true functional coefficient is $\beta_2(t, T)$ and the sample size is $N = 200$. The top row corresponds with the response variable following a Normal distribution while the bottom row shows its Poisson counterpart. The left column corresponds to the domain following a Uniform distribution and the right column represents the domain following a Negative Binomial distribution. The methods are from left to right: BE-VDFR, SOF, VDFR.

© 2025 WILEY-VCH Verlag GmbH & Co. KGaA, Weinheim

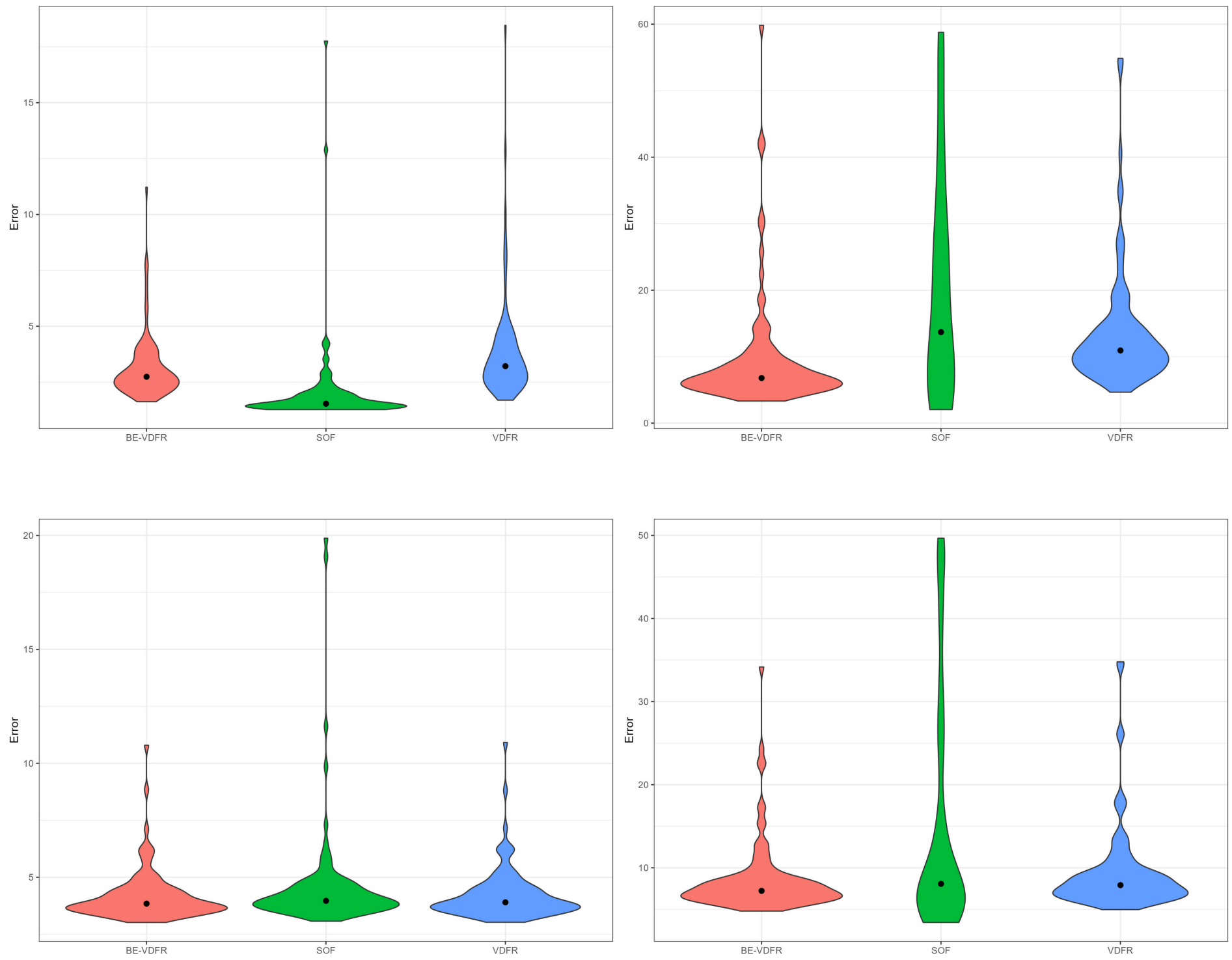

Figure 7 Violin box-plots of the RMSE for scenarios where the true functional coefficient is $\beta_3(t, T)$ and the sample size is $N = 200$. The top row corresponds with the response variable following a Normal distribution while the bottom row shows its Poisson counterpart. The left column corresponds to the domain following a Uniform distribution and the right column represents the domain following a Negative Binomial distribution. The methods are from left to right: BE-VDFR, SOF, VDFR.

© 2025 WILEY-VCH Verlag GmbH & Co. KGaA, Weinheim

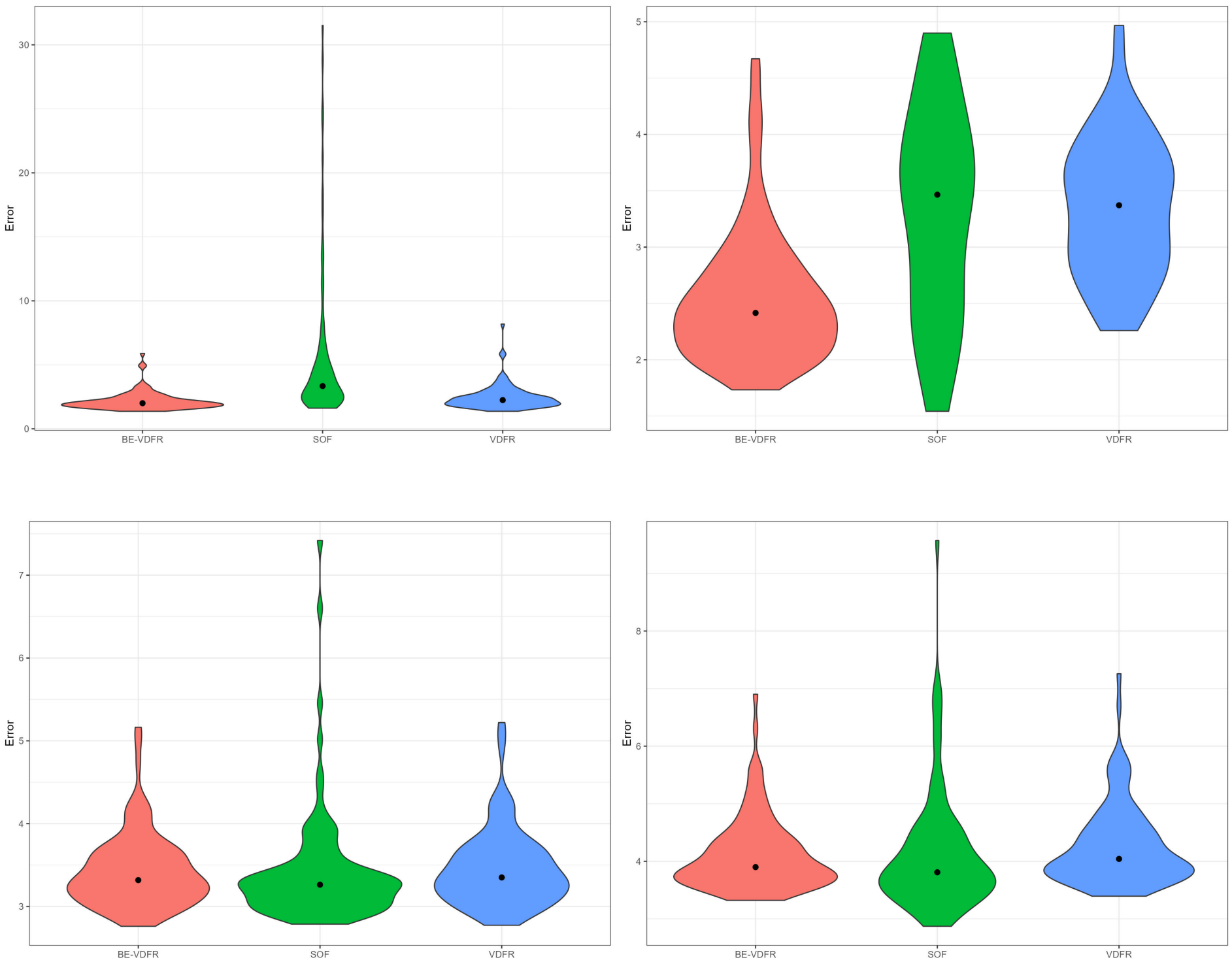

Figure 8 Violin box-plots of the RMSE for scenarios where the true functional coefficient is $\beta_4(t, T)$ and the sample size is $N = 200$. The top row corresponds with the response variable following a Normal distribution while the bottom row shows its Poisson counterpart. The left column corresponds to the domain following a Uniform distribution and the right column represents the domain following a Negative Binomial distribution. The methods are from left to right: BE-VDFR, SOF, VDFR.

1.3 AMSE Results for N=100

Figure 9 through Figure 12 present the Average Mean Square Error (AMSE) distributions for functional coefficient estimation with sample size $N = 100$.

© 2025 WILEY-VCH Verlag GmbH & Co. KGaA, Weinheim

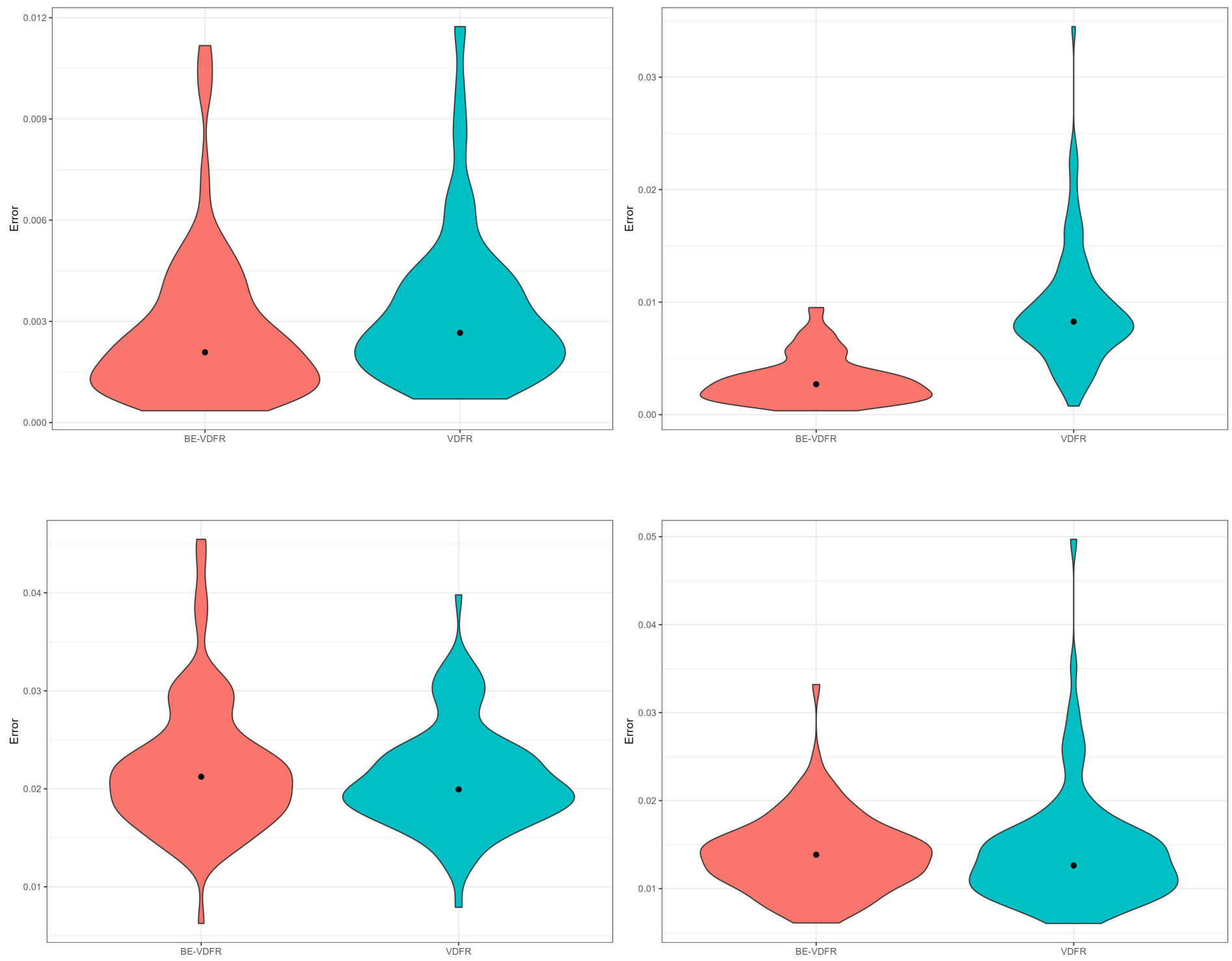

Figure 9 Violin box-plots of the AMSE for scenarios where the true functional coefficient is $\beta_1(t, T)$ and the sample size is $N = 100$. The top row corresponds with the response variable following a Normal distribution while the bottom row shows its Poisson counterpart. The left column corresponds to the domain following a Uniform distribution and the right column represents the domain following a Negative Binomial distribution. The methods are from left to right: BE-VDFR, VDFR.

© 2025 WILEY-VCH Verlag GmbH & Co. KGaA, Weinheim

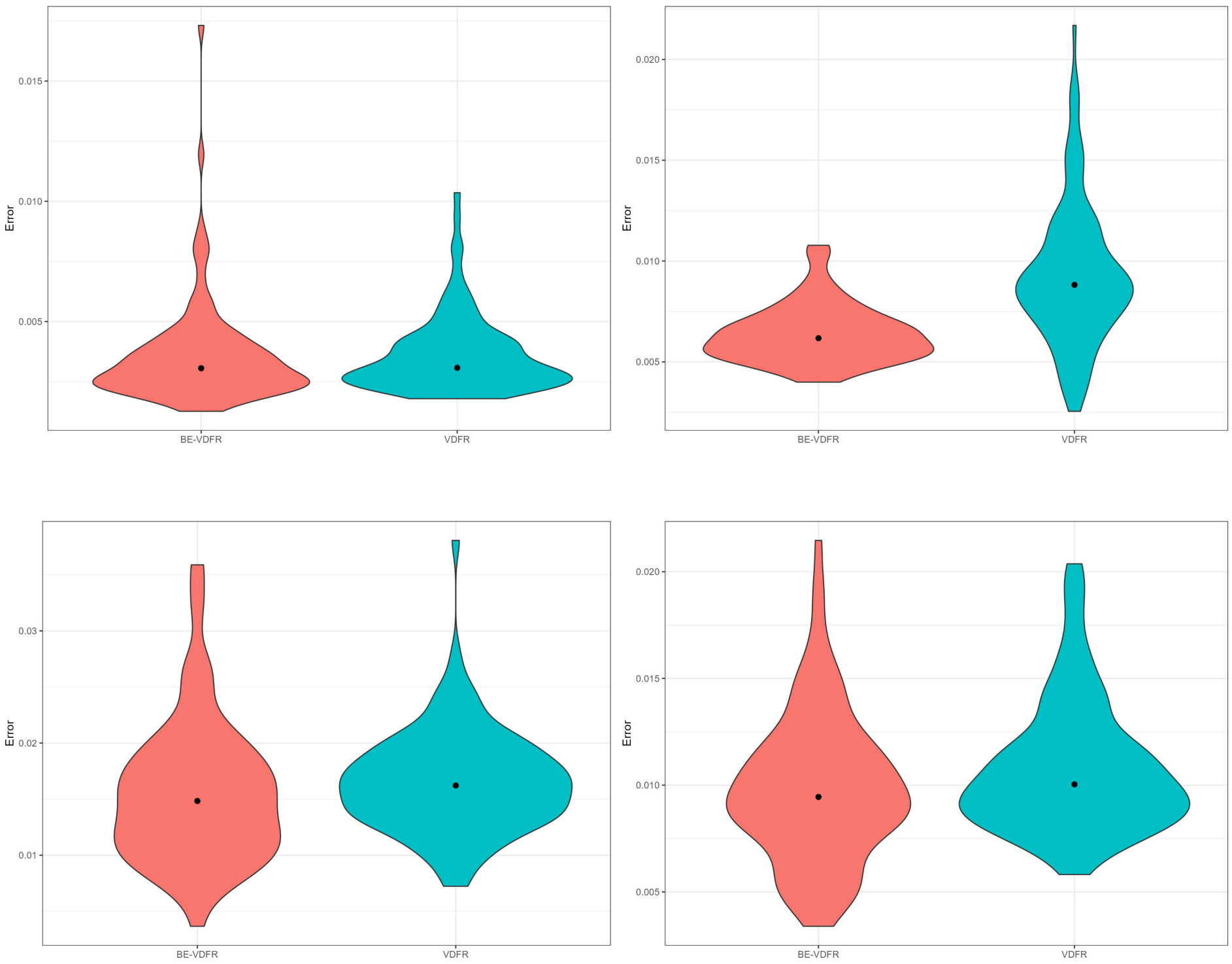

Figure 10 Violin box-plots of the AMSE for scenarios where the true functional coefficient is $\beta_2(t, T)$ and the sample size is $N = 100$. The top row corresponds with the response variable following a Normal distribution while the bottom row shows its Poisson counterpart. The left column corresponds to the domain following a Uniform distribution and the right column represents the domain following a Negative Binomial distribution. The methods are from left to right: BE-VDFR, VDFR.

© 2025 WILEY-VCH Verlag GmbH & Co. KGaA, Weinheim

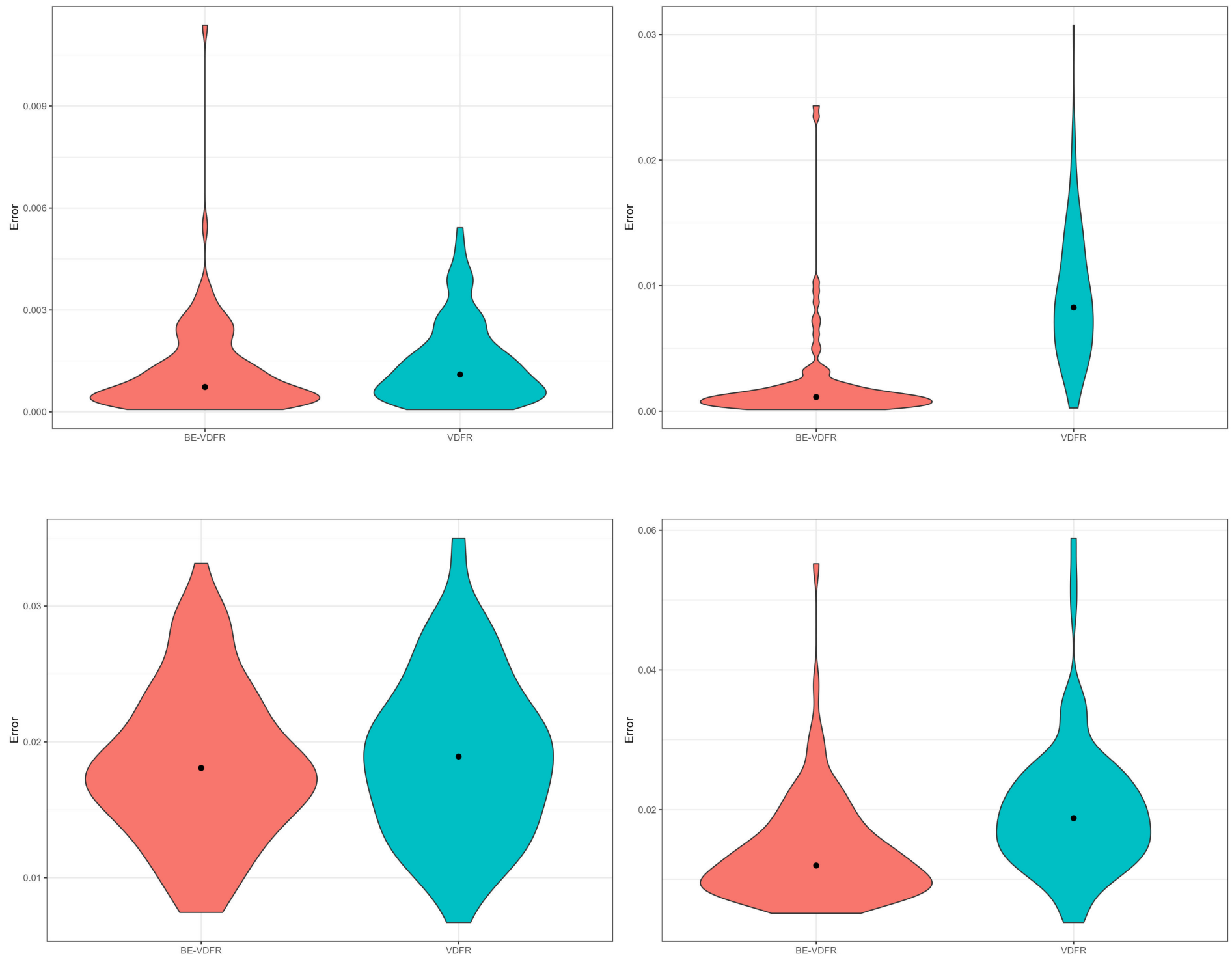

Figure 11 Violin box-plots of the AMSE for scenarios where the true functional coefficient is $\beta_3(t, T)$ and the sample size is $N = 100$. The top row corresponds with the response variable following a Normal distribution while the bottom row shows its Poisson counterpart. The left column corresponds to the domain following a Uniform distribution and the right column represents the domain following a Negative Binomial distribution. The methods are from left to right: BE-VDFR, VDFR.

© 2025 WILEY-VCH Verlag GmbH & Co. KGaA, Weinheim

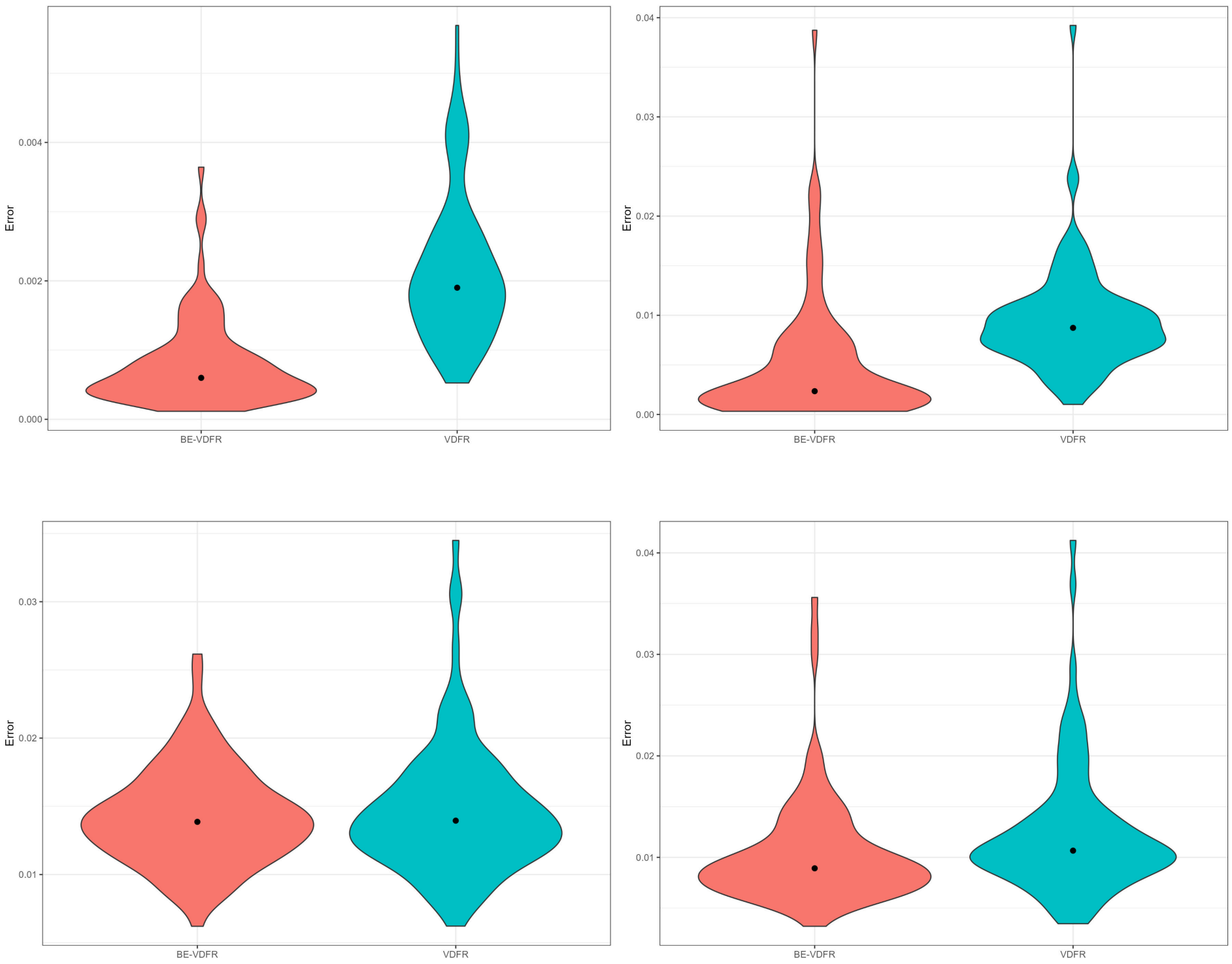

Figure 12 Violin box-plots of the AMSE for scenarios where the true functional coefficient is $\beta_4(t, T)$ and the sample size is $N = 100$. The top row corresponds with the response variable following a Normal distribution while the bottom row shows its Poisson counterpart. The left column corresponds to the domain following a Uniform distribution and the right column represents the domain following a Negative Binomial distribution. The methods are from left to right: BE-VDFR, VDFR.

© 2025 WILEY-VCH Verlag GmbH & Co. KGaA, Weinheim

1.4 AMSE Results for N=200

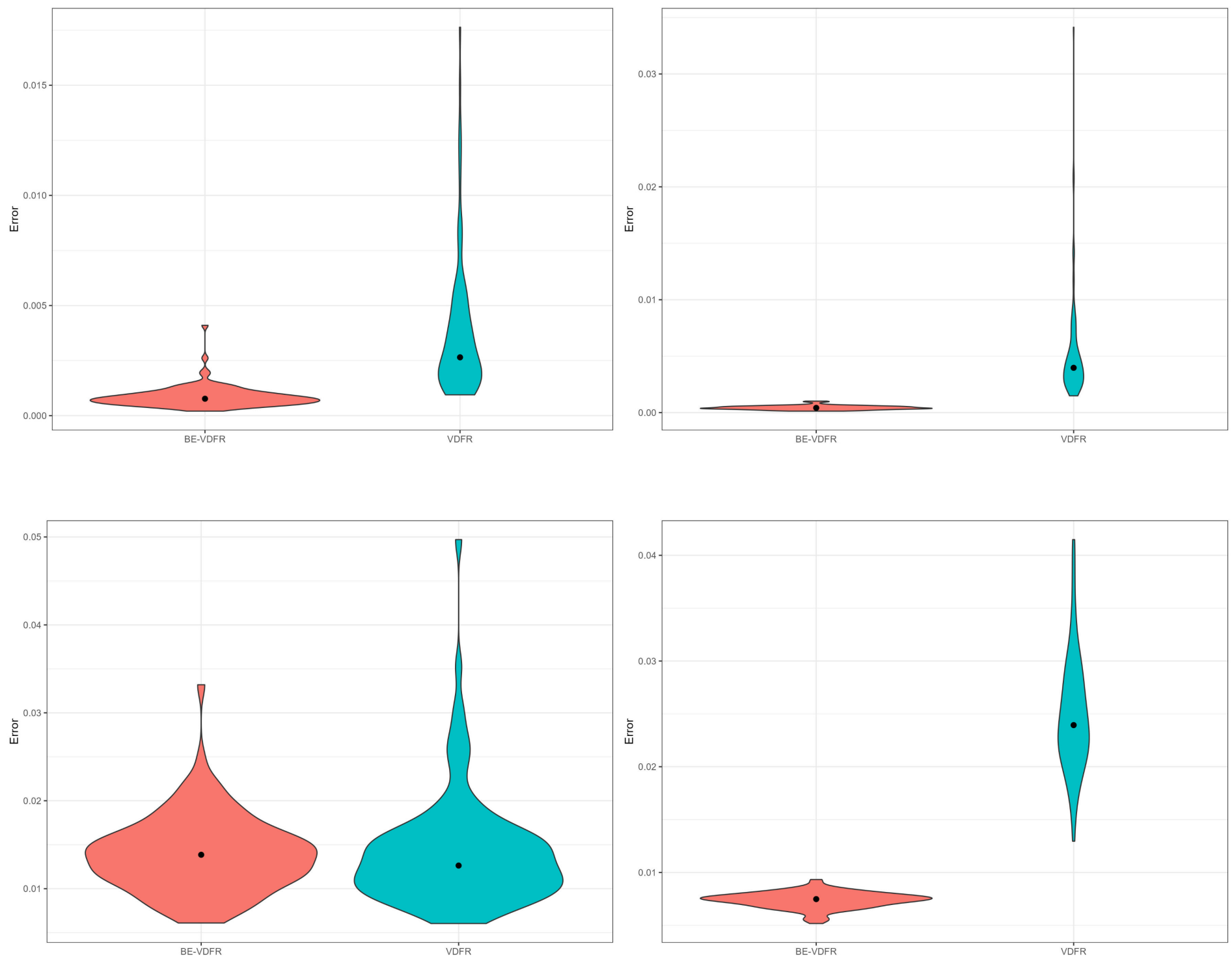

Figure 13 Violin box-plots of the AMSE for scenarios where the true functional coefficient is $\beta_1(t, T)$ and the sample size is $N = 200$. The top row corresponds with the response variable following a Normal distribution while the bottom row shows its Poisson counterpart. The left column corresponds to the domain following a Uniform distribution and the right column represents the domain following a Negative Binomial distribution. The methods are from left to right: BE-VDFR, VDFR.

© 2025 WILEY-VCH Verlag GmbH & Co. KGaA, Weinheim

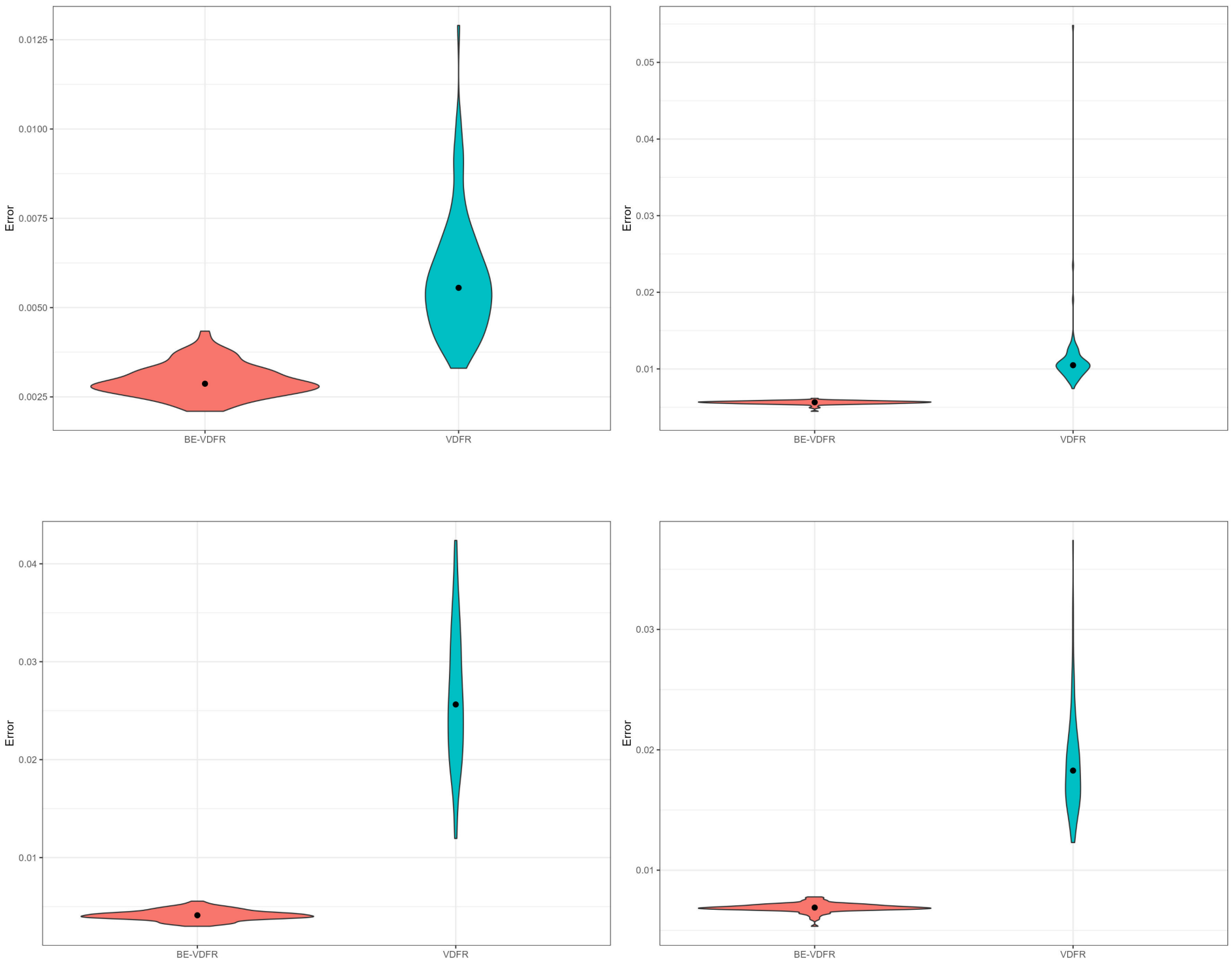

Figure 14 Violin box-plots of the AMSE for scenarios where the true functional coefficient is $\beta_2(t,T)$ and the sample size is $N = 200$. The top row corresponds with the response variable following a Normal distribution while the bottom row shows its Poisson counterpart. The left column corresponds to the domain following a Uniform distribution and the right column represents the domain following a Negative Binomial distribution. The methods are from left to right: BE-VDFR, VDFR.

© 2025 WILEY-VCH Verlag GmbH & Co. KGaA, Weinheim

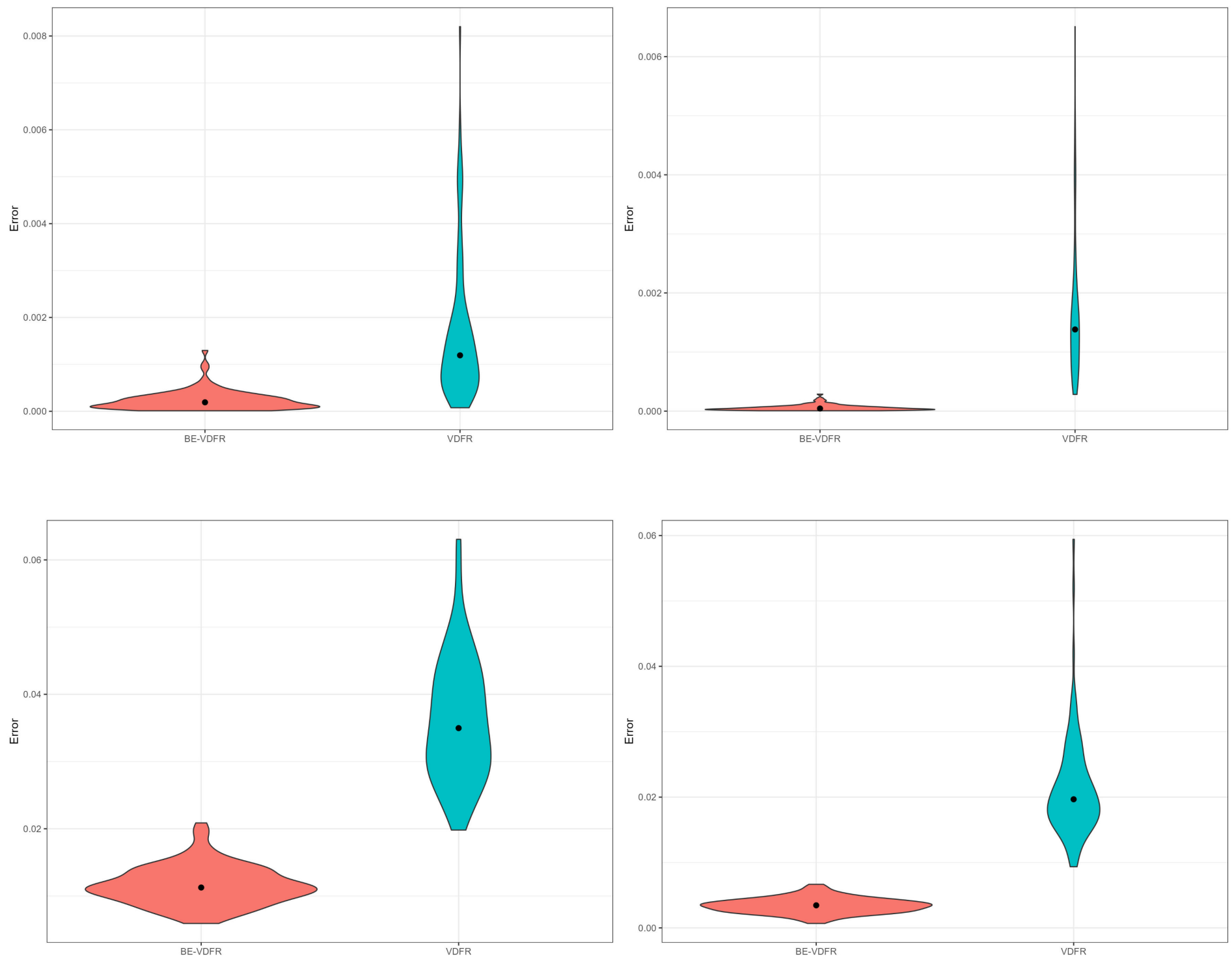

Figure 15 Violin box-plots of the AMSE for scenarios where the true functional coefficient is $\beta_3(t,T)$ and the sample size is $N = 200$. The top row corresponds with the response variable following a Normal distribution while the bottom row shows its Poisson counterpart. The left column corresponds to the domain following a Uniform distribution and the right column represents the domain following a Negative Binomial distribution. The methods are from left to right: BE-VDFR, VDFR.

© 2025 WILEY-VCH Verlag GmbH & Co. KGaA, Weinheim

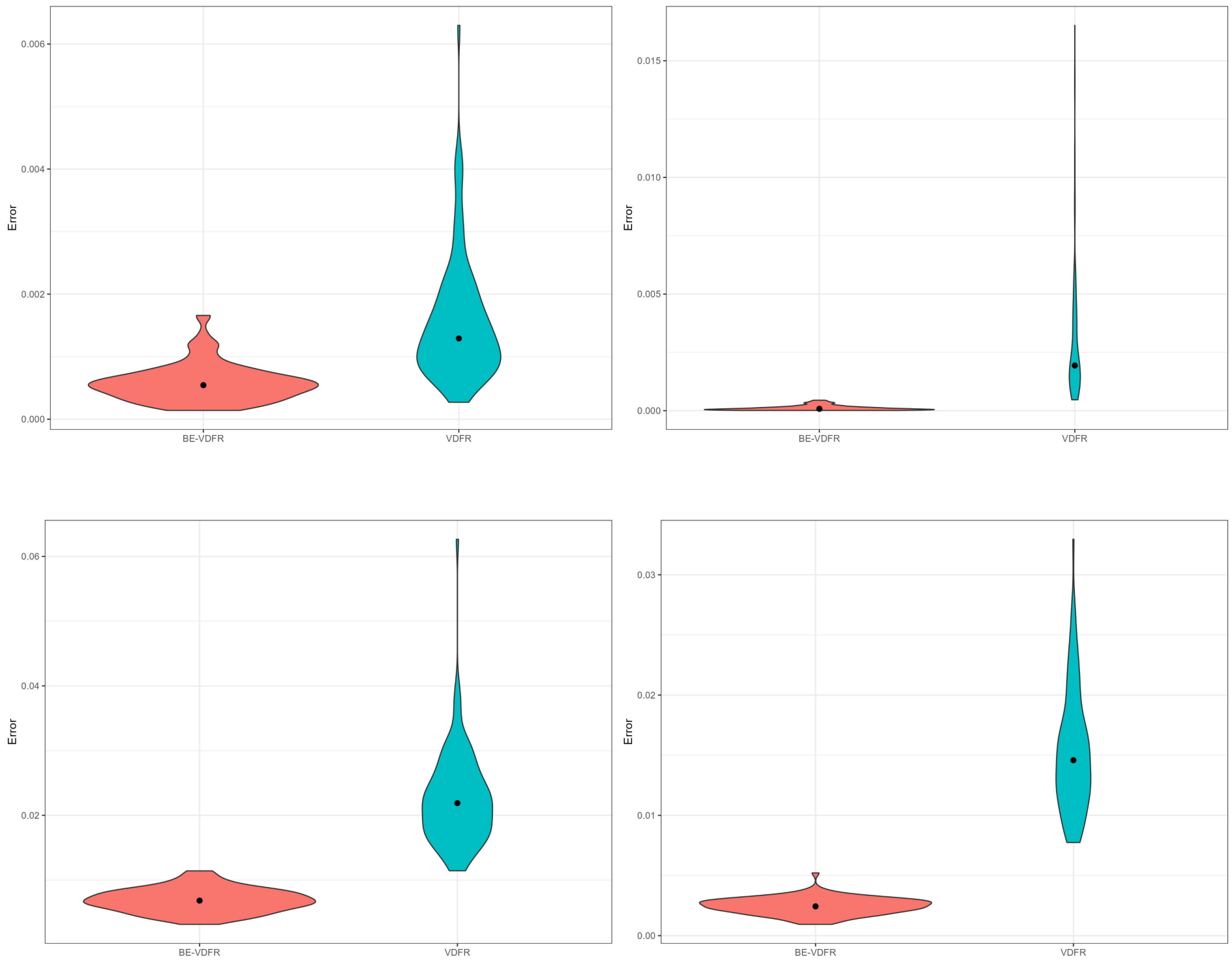

Figure 16 Violin box-plots of the AMSE for scenarios where the true functional coefficient is $\beta_4(t, T)$ and the sample size is $N = 200$. The top row corresponds with the response variable following a Normal distribution while the bottom row shows its Poisson counterpart. The left column corresponds to the domain following a Uniform distribution and the right column represents the domain following a Negative Binomial distribution. The methods are from left to right: BE-VDFR, VDFR.

© 2025 WILEY-VCH Verlag GmbH & Co. KGaA, Weinheim
